# Association of Tinnitus With Speech Recognition and Executive Functions in Older Adults

**DOI:** 10.1177/23312165251389585

**Published:** 2025-11-13

**Authors:** Nick Sommerhalder, Zbyněk Bureš, Oliver Profant, Tobias Kleinjung, Patrick Neff, Martin Meyer

**Affiliations:** 1 Evolutionary Neuroscience of Language, 31084 Institute for the Interdisciplinary Study of Language Evolution, University of Zurich, Zurich, Switzerland; 2 Department of Technical Studies, 251262 College of Polytechnics, Jihlava, Czech Republic; 3 Department of Otorhinolaryngology, Third Faculty of Medicine, Charles University, Prague, Czech Republic; 4 Department of Auditory Neuroscience, Institute of Experimental Medicine, Czech Academy of Sciences, Prague, Czech Republic; 5 Department of Otorhinolaryngology—Head and Neck Surgery, 87738 University Hospital of Zurich, University of Zurich, Zurich, Switzerland; 6 Department of Psychiatry and Psychotherapy, University of Regensburg, Regensburg, Germany; 7 Center for the Interdisciplinary Study of Language Evolution, University of Zurich, Zurich, Switzerland; 8 Neuroscience Center Zurich, University of Zurich, Zurich, Switzerland

**Keywords:** older adults, chronic subjective tinnitus, suprathreshold audiometry, speech comprehension, cognition, executive functions

## Abstract

Adults with chronic subjective tinnitus often struggle with speech recognition in challenging listening environments. While most research demonstrates deficits in speech recognition among individuals with tinnitus, studies focusing on older adults remain scarce. Besides speech recognition deficits, tinnitus has been linked to diminished cognitive performance, particularly in executive functions, yet its associations with specific cognitive domains in ageing populations are not fully understood. Our previous study of younger adults found that individuals with tinnitus exhibit deficits in speech recognition and interference control. Building on this, we hypothesized that these deficits are also present for older adults. We conducted a cross-sectional study of older adults (aged 60–79), 32 with tinnitus and 31 controls matched for age, gender, education, and approximately matched for hearing loss. Participants underwent audiometric, speech recognition, and cognitive tasks. The tinnitus participants performed more poorly in speech-in-noise and gated speech tasks, whereas no group differences were observed in the other suprathreshold auditory tasks. With regard to cognition, individuals with tinnitus showed reduced interference control, emotional interference, cognitive flexibility, and verbal working memory, correlating with tinnitus distress and loudness. It is concluded that tinnitus-related deficits persist and even worsen with age. Our results suggest that altered central mechanisms contribute to speech recognition difficulties in older adults with tinnitus.

## Introduction

Chronic subjective tinnitus is the constant perception of sounds that are perceived only by the person affected and occur without an external acoustic source (Eggermont & Roberts, 2004; Langguth et al., 2013). Tinnitus may be subdivided into “tinnitus” and “tinnitus disorder.” The latter term is used to describe cases of tinnitus accompanied by emotional disturbance and/or cognitive impairments and/or autonomic arousal, which can result in changes in behavior and reduced functionality (De Ridder et al., 2021). The prevalence of tinnitus is estimated to be around 10–15% of the population (Biswas et al., 2022; Cederroth et al., 2013; Jarach et al., 2022; McCormack et al., 2016). Recent studies indicate that approximately 1% to 4% of the population suffer severely from tinnitus (Biswas et al., 2022; Langguth et al., 2013).

A major risk factor associated with tinnitus is noise-induced or age-related hearing loss (Baguley et al., 2013; Nelson & Chen, 2004), although the relationship between tinnitus and hearing loss is complex. Many individuals with hearing loss do not experience tinnitus, and conversely, not all people with tinnitus exhibit clearly measurable hearing loss (Guest et al., 2017; Liberman & Liberman, 2015; Schaette & McAlpine, 2011). In addition to hearing loss, age has emerged as an independent risk factor for tinnitus. Although the increased prevalence of tinnitus in older adults is often attributed to age-related hearing loss (presbycusis), recent studies suggest that ageing per se may also play a role in tinnitus onset (Reisinger et al., 2023a; 2023b).

Given that tinnitus prevalence increases with age (Jarach et al., 2022; McCormack et al., 2016), it is important to focus research efforts on older adults with tinnitus. This includes examining not only the relationship with various kinds of hearing loss but also exploring other factors that can be affected by age such as cognitive functions.

### Tinnitus and (Suprathreshold) Hearing

Recent tinnitus research has expanded beyond the peripheral auditory system to investigate more central auditory factors. The central auditory system's functional integrity plays a critical role in ensuring accurate suprathreshold perception of stimuli presented at levels above the hearing threshold and speech-in-noise (SiN) recognition (Giroud et al., 2018). To assess suprathreshold perception, researchers employ various tasks, including, for example, gap detection tasks to evaluate the ability to perceive rapid temporal changes (He et al., 1999), modulation tasks to measure sensitivity to amplitude and frequency fluctuations (Joris et al., 2004), and intensity discrimination tasks to assess the ability to perceive differences in intensity between sounds (Carlyon & Moore, 1984). It is presumed that these functions are mediated by neural structures in the auditory pathway and the cerebral cortex rather than in the cochlea.

Research on gap detection, modulation, and intensity discrimination using tinnitus and control participants shows mixed results. For gap detection, some studies reported poorer performance for tinnitus participants (Fournier & Hébert, 2013; Gilani et al., 2013; Ibraheem & Hassaan, 2017), while others found no differences (An et al., 2014; Boyen et al., 2015; Kenneth & Werff, 2019; Zeng et al., 2020). Modulation detection results are similarly inconsistent, some noted poorer performance for tinnitus participants (Paul et al., 2017), and others reported no group differences (Bureš et al., 2019). For intensity discrimination, Epp et al. (2012) reported poorer performance in tinnitus participants. In contrast, Bureš et al. (2019) and Zeng et al. (2020) found that tinnitus participants outperformed controls. In Zeng et al. (2020), this advantage was limited to specific measurement conditions and did not extend uniformly across all tested frequencies.

The limited and often contradictory research in this field makes it challenging to draw definitive conclusions about the impact of tinnitus on suprathreshold auditory processing. This is particularly true for older adults.

### Tinnitus and Speech Recognition

The findings for speech recognition reveal a more clear pattern compared to the results for suprathreshold auditory perception. SiN scenarios are often used to analyze the effect of background noise on speech recognition. In SiN scenarios, individuals with tinnitus often perform more poorly than controls (e.g., Gilles et al., 2016; Ivansic et al., 2017; Madhukesh et al., 2024; Niewiarowicz et al., 2022; Vielsmeier et al., 2016). Notably, these impairments have been observed even for individuals with normal audiometric thresholds. This reflects the common complaint of individuals with tinnitus, namely difficulties in understanding speech in noisy environments (Andersson et al., 2001; Sanchez & Stephens, 2000).

Despite the weight of evidence, some studies have not replicated SiN deficits in individuals with tinnitus (e.g., Bureš et al., 2019; Zeng et al., 2020). Zeng et al. (2020) used a task with background noise of either white noise or a single talker, which may partly account for the discrepancy in findings, as different types of background noise can differentially affect speech recognition performance. Multitalker babble produces stronger informational masking than white noise or a single competing talker (Corbin et al., 2016) and may therefore be more sensitive to tinnitus-related deficits in speech recognition.

Bureš et al. (2019) focused their investigation on older adults with tinnitus. Although their results showed a trend in the expected direction, they did not find statistically significant differences in performance between individuals with tinnitus and controls.

While the majority of studies have focused on (younger) adults, only a few have explored tinnitus and speech recognition for older populations, such as Bureš et al. (2019) and Oosterloo et al. (2020). Oosterloo et al. (2020) used data from the retrospective population-based Rotterdam Study and showed poorer SiN recognition for older adults with tinnitus compared to controls, but only for those with hearing loss. To date only a few studies with conflicting results have focused on older adults.

### Tinnitus and Cognition

Cognitive functions, particularly executive functions (EF; a set of processes essential for concentration, focused attention, and navigating situations where automatic responses are inadequate; Diamond, 2013) play a crucial role in speech recognition (Arlinger et al., 2009). A particular aspect of EFs, namely working memory, referring to the cognitive capacity to retain and manipulate sensory information over short periods of time (Diamond, 2013), has been identified as an important cognitive predictor of SiN recognition (Akeroyd, 2008; Rönnberg et al., 2013). This relationship may be age-dependent, as, for example, working memory seems less important for SiN performance in younger adults (Füllgrabe et al., 2015).

The relationship between SiN performance and cognition is important, as ageing is associated with increased tinnitus, hearing loss, and cognitive decline (Ferri et al., 2005; Flier & Scheltens, 2005; Lobo et al., 2000). Hearing loss is a significant risk factor for cognitive impairment (Loughrey et al., 2018). Moreover, some studies suggest that tinnitus may be an independent risk factor for cognitive impairment or Alzheimer's disease (Chu et al., 2020; Jafari et al., 2019; Zhang et al., 2022).

Several meta-analyses and reviews indicate that (young) adults with tinnitus show reduced cognitive performance, especially in EFs (Clarke et al., 2020; Mohamad et al., 2016; Sherlock & Brungart, 2021; Tegg-Quinn et al., 2016; Trevis et al., 2018). Numerous studies have shown that individuals with tinnitus show poorer working memory than controls (Nagaraj et al., 2020; Rossiter et al., 2006; Sharma et al., 2023; Trevis et al., 2016). Similarly, most studies report inferior performance in the domain of interference control for individuals with tinnitus compared to controls (Araneda et al., 2015, 2018; Gonendik et al., 2021; Stevens et al., 2007). Interference control refers to the cognitive ability to suppress dominant responses in the presence of conflicting information (Nigg, 2000). Moreover, deficits in cognitive flexibility (encompassing the ability to adapt one's perspective or approach to problem solving, adjust to new demands or rules, and shift between tasks; Diamond, 2013) have been identified in tinnitus (Cardon et al., 2019; Chen et al., 2018; Stevens et al., 2007). In addition to the observed deficits in EFs, researchers have identified impairments in selective and/or sustained attention among individuals with tinnitus compared to controls (Rossiter et al., 2006; Stevens et al., 2007).

While most of this research has been done in (younger) adults, there only are a few studies of specific cognitive functions for older adults with tinnitus. Hamza and Zeng (2021) conducted an analysis of data for older adults, revealing unexpected superior cognitive performance among non-Hispanic individuals with both tinnitus and hearing loss. However, due to methodological limitations, such as the retrospective nature and the combination of several cognitive test into one factor, the interpretation of these results warrants caution. Finally, there are some studies that do not support cognitive deficits in (younger) adults with tinnitus (e.g., Andersson et al., 2009; Waechter et al., 2019, 2021).

When observed, cognitive deficits for individuals with tinnitus appear to be modality specific. For instance, Araneda et al. (2018) demonstrated that individuals with tinnitus performed more poorly than controls in Stroop tasks, with more pronounced deficits in the auditory-administered version than the visual version. Gonendik et al. (2021) reported similar findings, further supporting the notion of modality-specific cognitive impairments for those with tinnitus.

In summary, numerous studies provide evidence of cognitive deficits for individuals with tinnitus, particularly for EFs, and these impairments may be modality-specific. However, there are hardly any studies on older adults with tinnitus.

### Research Questions

Our research was intended to address critical gaps in the literature by focusing on older adults. We thoroughly investigated speech recognition and cognition for older individuals with and without tinnitus. Building upon previous studies, including our recent study (Sommerhalder et al., 2023), we posit that older individuals with tinnitus will show poorer speech recognition in challenging listening environments (SiN task and gated speech task), while elementary/suprathreshold auditory perception will not be affected. In the realm of cognitive functions, we anticipate that individuals with tinnitus will show inferior performance across a range of EFs primarily interference control, cognitive flexibility, and verbal working memory, but also selective/sustained attention (Stroop and Emotional Stroop task, trail making test, phonemic fluency task, digit span task backwards, d2-R task). We further anticipate that deficits in individuals with tinnitus exhibit a modality-specific pattern, with significantly more pronounced impairments in the auditory/verbal domain compared to the visual–spatial/nonverbal domain (digit span backwards task vs. Corsi block span backwards task, phonemic fluency task vs. design fluency task, and divided attention task auditory modality vs. visual modality), a pattern that may be further exacerbated by ageing. Such findings would suggest that tinnitus itself interacts with cognitive systems, particularly diminishing performance on tasks with auditory content.

## Materials and Methods

### Participants

We recruited a total of 68 Swiss-German-speaking participants between the ages of 60 and 79, 33 with tinnitus (tinnitus group, TI) and 35 without (control group, CG). All participants in the tinnitus group had chronic subjective tinnitus that had persisted for at least 24 months. Inclusion criteria included a pure-tone average between 10 and 40 dB HL (mean across 0.5, 1, 2, and 4 kHz). We excluded one participant from the tinnitus group and one from the CG due to profound hearing loss. We further excluded two CG participants due to exceptionally good hearing. Retaining these two CG participants in the sample diminishes the quality of matching between the two groups but does not substantially alter the observed effects of our analyses. Additionally, we excluded one participant from the CG due to asymmetrical hearing loss (greater than 20 dB HL difference in PTA across ears). The final sample included 32 participants (14 female) in the tinnitus group and 31 participants (15 female) in the CG. All participants reported being free from neurological conditions and psychological disorders. None were professional musicians, had formal musical training beyond school music programs, or spent more than 2 hr/week playing a musical instrument. We recruited participants from the Interdisciplinary Tinnitus Research Zurich Group's participant pool, the Department of Psychology's participant pool, advertisements in the Senior Citizens University of Zurich (UZH3), the Healthy Longevity Center of the University of Zurich, and Pro Senectute. The Ethics Committee of the Faculty of Arts and Social Sciences at the University of Zurich approved the study (Permit No. 21.4.18 and No. 22.4.9), and all participants provided written informed consent. Volunteers were paid for their participation.

### Procedure

Each participant attended a 2–3-hr appointment. Prior to the laboratory visit, tinnitus participants completed tinnitus-specific questionnaires. Upon arrival, all participants signed consent forms, filled out a health questionnaire, and completed the Montreal Cognitive Assessment (MoCA) (Nasreddine et al., 2005). For the tinnitus group, the first part of the tinnitometry (audiometric measurement of tinnitus parameters such as pitch, loudness) assessment followed. Subsequently, participants completed the audiometric and cognitive assessments. The order of the assessments was randomized, and during each assessment, two versions with different task sequences were also randomized to reduce order effects. After the first part, individuals with tinnitus completed the second part of the tinnitometry. Then, the second part of either the audiometric or cognitive tasks followed.

### Questionnaires

Five questionnaires were used to assess various health factors and psychopathological aspects. All participants completed a questionnaire on demographics and general health. We used the Beck Depression Inventory 2 (BDI) to assess depressive symptoms (Beck et al., 1996; Hautzinger et al., 2009) and the Geräuscheüberempfindlichkeits-Fragebogen (GÜF) for hyperacusis symptoms (Nelting & Finlayson, 2004). The tinnitus group completed the Tinnitus Handicap Inventory (THI) (Kleinjung et al., 2007; Newman et al., 1996), the Tinnitus Sample Case History Questionnaire (Langguth et al., 2007a), and three visual analog scales (VAS; 11-point, ranging from 0 to 10; for subjective perception of tinnitus loudness, annoyance, and ignorability; Langguth et al., 2007a).

### Audiometry

Audiometric testing was conducted in a sound-insulated audiometric chamber, with a specialized measurement system. The device and software were developed at the Institute of Experimental Medicine, Czech Academy of Sciences (Bureš et al., 2019; Profant et al., 2019). The audiometric device incorporates a high-quality audio interface (RME Fireface, RME, Germany) with a programable attenuator. The custom software to control the device was built in Matlab (Mathworks Inc., Natick, MA, USA). The testing setup included Sennheiser High-Frequency Audiometric Headsets HDA 300 (Sennheiser, Germany) and an Arturia BeatStep controller (Arturia, France). The equipment was calibrated in accordance with ISO 389–5, ISO 389–8, ISO 8253–3, and IEC 60645–3 standards, using the Brüel and Kjær artificial ear 4153. Table 1 presents an overview of the parameters and procedures employed for each audiometric test.

**Table 1.** Auditory Tests.

Test	Description	Stimuli	Participant task	Procedure	Threshold	Reference
PTA	Pure-tone audiograms	Pure-tones (0.125, 0.25, 0.5, 1, 2, 4, 6, and 8 kHz)	Keypress when tone is perceived	Response: level decreased by 4 dB; no response: level increased by 2 dB	PTA was calculated as the average hearing threshold (in dB HL) at frequencies of 0.5, 1, 2, and 4 kHz.	Bureš et al. (2019)
PTAnoise	Pure-tone audiograms with background white noise	Pure-tones (0.5, 1, 2, 4, 6, and 8 kHz), background white noise (60 dB SPL)				
GAP	Detect fast temporal changes	White noise, three gaps in white noise (ISI: 150 ms)	Keypress when gaps are perceived	Response: gap duration decreased by 1 ms; no response: gap duration increased by 1 ms	Gap length at 50% detection threshold	Bureš et al. (2019); He et al. (1999)
AM	Sensitivity to amplitude modulation	Two tones: one amplitude modulated (1 kHz carrier frequency, duration 2 s each, ISI: 200 ms, ITI: 2 s); modulation frequencies: 2, 4, 8 Hz)	Keypress (1 of 2) to indicate modulated tone	Correct response: modulation index halved; wrong response: modulation index doubled	Modulation index estimates for 2, 4, and 8 Hz	Bureš et al. (2019); Joris et al. (2004)
FM	Sensitivity to frequency modulation	Two tones: one frequency modulated (1 kHz carrier frequency, duration 2 s each, ISI: 200 ms, ITI: 2 s); modulation frequencies: 2, 4, 8 Hz)				
DLI	Smallest audible level difference	Two tones: one louder (1 kHz, duration 500 ms each, ISI: 200 ms, ITI: 2 s).	Keypress (1 of 2) to indicate louder tone	Correct response: level difference halved; wrong response: level difference doubled	Level difference estimate (dB)	Bureš et al. (2019); Carlyon and Moore (1984); Köning & Lüscher (1962)
FPT	Auditory temporal sequencing and pattern recognition	Three consecutive tones with two frequencies (low: 880 Hz, high: 1122 Hz, ISI: 200 ms, ITI: 5 s)	Repeat pattern of pitches (e.g., “high,” “low,” “low”)	Sets of three different tones were played	Correct responses (in %)	Musiek (1994)
DPT	Temporal coding	Three consecutive tones with two durations (short: 250 ms, long: 500 ms, 1 kHz, ISI; 200 ms, ITI: 5 s)	Repeat pattern of durations (e.g., “long,” “short,” “long”)			
						
SRT	Speech recognition threshold	Sets of 10 words: 4 one-syllable, 4 two-syllable, 2 three-syllable; female speaker.	Repeat words	First set presented at 40 dB; level increased in 10 dB steps until >50% word recognition	Level (dB) at 50% detection threshold	Seeman (1960)
SIN	Speech comprehension in babble noise	Sets of 10 sentences (average length: 11.62 words, female speaker, unpredictable, natural); babble noise: mix of six independent male and female voices, unintelligible	Repeat sentences	More than 50% correctly identified: noise increased by 5 dB; otherwise decreased by 5 dB	Signal-to-noise ratio (dB) at 50% detection threshold	Bureš et al. (2019); Dlouhá et al. (2011)
GS	Recognition of gated (interrupted) speech			Initially duty cycle set to 30% then increased to 55% and then to 70%	Duty cycle (in %) at 50% detection threshold	Bureš et al. (2019)

*Note.* Each auditory test, along with the respective parameters and procedures, is listed in the table. AP = adaptive procedure; WN = white noise; PTA = pure-tone average; GDT = gap detection task; AM = amplitude modulation; FM = frequency modulation; DLI = difference limen for intensity; FPT = frequency pattern test; DPT = duration pattern test; SRT = speech recognition threshold; SiN = speech-in-noise; GS = gated speech; ISI = interstimulus interval; ITI = intertrial interval; CF = carrier frequency.

### Cognition

To assess cognitive function, we administered seven standard psychometric tests in a quiet, one-on-one setting. Table 2 outlines the procedures and parameters for each cognitive test. For the divided attention task, the subtest “Divided Attention” from the computerized “Testbatterie zur Aufmerksamkeitsprüfung” version 2.3.1 was used (Zimmermann & Fimm, 2017).

**Table 2.** Cognitive Tests.

Task	Domain	Stimuli	Participant task	Evaluation	Threshold value	Reference
Stroop task	Processing speed	Part A: 24 colored dots, arranged in six rows with four elements of each color (gelb/yellow, rot/red, blau/blue, grün/green)	Name the color of colored dots	Time in seconds to name the elements	Time in seconds	Spreen and Strauss (1998)
	Interference control	Part C: 24 color words written in a different color, arranged in six rows with four elements of each color	Name the color of color words		Ratio of completion time for Part C to Part A (in seconds)	
	Emotional interference	Part B: 24 neutral words written in color (wenn/when, kaum/hardly, und/and, oben/above); Part D: 24 tinnitus-words written in color (schrill/shrill, pfeifen/whistle, zischen/hiss, laut/loud)	Name the color of the words		Subtracted the time from part B from the time in part D	Dresler et al. (2009)
Trail making test	Processing speed	Part A: 25 ascending numbers in circles	Connect numbers in ascending order	Time in seconds to connect elements with a pen.	Time in seconds	Tombaugh (2004)
	Cognitive flexibility	Part B: 13 numbers and 12 letters in circles	Connect numbers and letters alternately in ascending respectively alphabetical order		Ratio of completion time for Part B to Part A (in seconds)	
Phonemic fluency	Verbal initiation		Generate as many words as possible with a given initial letter	Count of unique words	The number of words produced within 2 min	Lezak (2004)
5-point test	Nonverbal initiation	Sheet with 40 squares, each containing five symmetrically arranged dots	Connect two or more dots with straight lines to create unique designs	Count of unique designs	The number of unique designs produced in 3 min	Regard et al. (1982)
Digit and Corsi Block Span	Verbal short-term memory	Sequences between three and eight digits, read-out by the examiner	Repeat the read-out numbers in the same order	As soon as two sequences of the same length were repeated incorrectly, the test was discontinued	Longest sequence correctly recalled	Wechsler (1987)
	Verbal working memory	Sequences between two and seven digits, read-out by the examiner	Repeat the read-out numbers in reverse order			
	Visuo-spatial short-term memory	Sequences between two and seven blocks on a board with nine blocks, tapped by the examiner	Tap the blocks in the same order as shown			
	Visuo-spatial working memory		Tap the blocks in reverse order as shown			
d2-R	Attention and concentration capacity	The letters d and p, arranged in 14 rows of 57 characters each, with 1–4 hyphens at the top and/or bottom	Cross out as many d's with two hyphens at the top and/or bottom, as possible	20 s per line; 14 lines in total	Count number of correct answers and errors	Brickenkamp et al. (2010)
Divided-attention task	Divided attention	Visual stimuli: field of 4 × 4 dots. There are always between six and eight crosses on this field, which change every 2 s; Auditory stimuli: alternating between a high tone and a low tone, at 1 s intervals	Visual task: press the button when four crosses are adjacent to each other and form a square; Auditory task: press the button when you hear the same sound twice in succession	Reaction time and mistakes	Reaction time and mistakes for visual stimuli; Reaction time and mistakes for auditory stimuli	Zimmermann and Fimm (2017)

*Note.* Each cognitive test, along with the respective parameters and procedures, is listed in the table.

### Tinnitometry

Our tinnitometry protocol involved tinnitus loudness and pitch matching. This process required participants to adjust a tone or narrowband noise to match their individual tinnitus perception as well as possible (Henry et al., 2004, 2006; Neff et al., 2019; Tyler & Baker, 1983). For participants with pure-tone-like tinnitus, we employed the “Match Your Tinnitus” app on a Huawei MediaPad M5 tablet (Huawei, China; Android version 8.0.0). Participants first adjusted the frequency of a tone to match the pitch of their tinnitus, and then set the loudness accordingly. For those with noise-like tinnitus, we used custom MAX 7 software (Cycling ‘74, USA) in combination with a Palette Expert Kit controller (Palette, Canada). In this condition, participants had to adjust the center frequency of the narrowband noise, followed by its bandwidth, and finally the loudness. In both cases, participants used Sennheiser HD 25 headphones (Sennheiser, Germany).

After participants completed the initial pitch and loudness adjustments, we administered a manual octave confusion test to determine whether their perceived tinnitus pitch more closely matched a tone or a narrow-band noise with center frequency one octave higher or lower than the initially selected frequency (Graham & Newby, 1962; Vernon et al., 1980). If the participant showed evidence of octave confusion (i.e., they selected the octave-shifted tone as a better match), we repeated the matching procedure and subsequently performed another octave confusion test to confirm the final pitch match.

### Statistical Analyses

For the statistical analyses we used Version 4.2.1 of the statistical software R (R Project, Vienna, Austria) and Rstudio Version 2023.12.1 (Posit PBC, Boston, United States) along with the packages “car,” “corrplot,” “dplyr,” “emmeans,” “fastDummies,” “ggplot2,” “lmtest,” “performance,” “ppcor,” “psych,” “pwrss,” “sjPlots,” “tidyverse,” and “visreg.” We defined statistical significance as p< .05 and reported trends at p< .1.

To estimate the required sample size, we conducted a priori power analysis using the R package “pwrss.” This analysis was based on the outcome measures of our previous study (Sommerhalder et al., 2023), at an alpha level of .05 and a desired power of .80. The power analysis indicated that a total sample size of at least 44 participants would be necessary. To account for potential attrition, exclusions, and enhance robustness of our findings, we aimed to recruit a sample 1.5 times larger than the minimum required sample size.

#### Main Models

Two main models where constructed to test our hypotheses. Both were ANCOVA models to control for age, gender, education (in years), and PTA.

Model 1: variable∼group+age+gender+education+PTA

– Model 1.1: $variable∼group+age+gender+education+PTA_{widerange}$

– Model 1.2: $variable∼group+age+gender+education+PTA_{4,6,8kHz}$

Model 2: score∼group*task_type+error(participant/task_type) + age+gender+education+PTA

For the group comparison we used Model 1. To analyze modality specific differences we used Model 2, with an interaction term between group and task type. There were some differences in the higher frequencies of the pure-tone audiograms between our two groups, which although not significant, could affect speech recognition. Therefore, we also ran Models 1.1 and 1.2, which included both wide range PTA ${}_{widerange}$ (0.125, 0.25, 0.5, 1, 2, 4, 6, and 8 kHz) and PTA ${}_{4,6,8kHz}$ for higher frequencies (4, 6, and 8 kHz). To further examine this issue, we conducted a mixed-design ANOVA with group as a between-subjects factor and frequency and ear as within-subjects factors, using hearing thresholds as the dependent variable. The results of this additional analysis are provided in the Supplemental Materials.

We checked the assumptions of the ANCOVA models with visual and analytical methods, see Supplemental Table S1. When the assumptions were not met we used a nonparametric rank-based ANCOVA-like alternative (Olejnik & Algina, 2016). Outliers were defined by a z-score greater than three. To adjust for multiple comparisons within each hypothesis we applied Bonferroni correction (auditory tasks and cognitive tests were both corrected for 14 comparisons). For every model we report the effect size partial $\eta^{2}$ for the group difference.

#### Secondary Analyses

We conducted exploratory statistical analyses within the tinnitus group to further examine relationships among tinnitus-related variables and the outcomes from the group comparisons. We calculated a bivariate Spearman correlation matrix, including these outcome variables together with tinnitus-related variables (THI scores, tinnitus loudness, and tinnitus duration in months, tinnitus loudness matching) and other covariates. We also examined the correlations between the THI or self-reported loudness (VAS) scores and the speech audiometry and cognitive scores.

Model 3: variable∼THI+age+gender+education+PTA

For this we computed Model 3 using speech and cognitive variables as dependent variables and THI scores as a predictor variable. Additionally, we computed a bivariate Spearman correlation matrix with (selected) demographic, auditory, speech, and cognitive variables for all participants and only participants with tinnitus: see Supplemental Figures S1 and S2.

## Results

### Participants’ Characteristics

**Figure 1.**
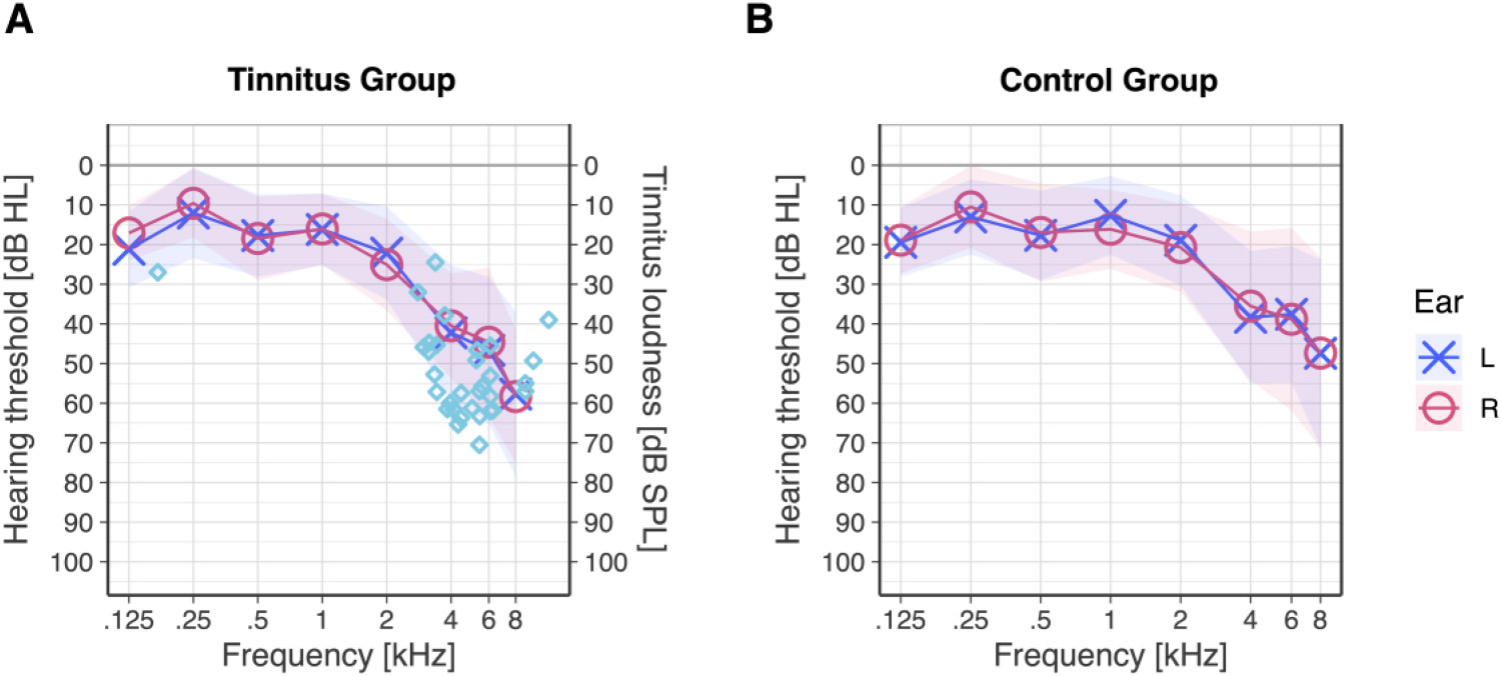
Mean pure-tone audiograms for both the tinnitus group and control group, alongside tinnitus pitch and loudness measurements. Colored ribbons represent one standard deviation around the mean for each group. (A) Mean audiogram for the tinnitus group. Turquoise-colored diamonds indicate individual tinnitus pitch and loudness estimates derived from the matching procedure. (B) Mean audiogram of the control group.

We attempted to match demographic and audiometric characteristics between the tinnitus group and the CG to minimize confounding variables. The two groups showed comparable mean ages (TI: 67.4 years, SD = 5.0; CG: 68.0 years, SD = 5.2), with no differences ( W = 458.0, p = .605). Gender ( W = 519.0, p = .720), education ( t = −0.8, p = .403), MoCA scores ( W = 459.0, p = .607), and BDI scores ( W = 481.5, p = .845) were also similar between the groups, for more details see Table 3. Average PTA values were similar, although not perfectly matched (TI: 24.8 dB HL, SD = 6.8; CG: 22.1 dB HL, SD = 7.9), but with no differences ( t = 1.5, p = .151), see Figure 1. Only two frequencies in the high-frequency range were different between the two groups, see Supplemental Table S2.

**Table 3.** Participants’ Characteristics.

	Tinnitus group					Control group					
	*M* ± *Stdv*	*Mdn*	Min	Max		*M* ± *Stdv*	*Mdn*	Min	Max		*p*
Gender (f/m/d)	14/18/0					15/16/0					.720
Age	67.4 ± 5.0	67.0	60.0	79.0		68.0 ± 5.2	66.0	60.0	77.0		.605
Education (yrs)	15.3 ± 3.3	14.0	9.0	24.0		15.9 ± 2.6	16.0	12.0	22.0		.403
PTA (dB HL)	24.8 ± 6.8	24.7	12.4	34.8		22.1 ± 7.9	20.0	10.8	38.2		.151
MoCA	28.1 ± 1.4	28.0	26.0	30.0		28.2 ± 1.1	28.0	26.0	30.0		.607
BDI	3.3 ± 4.1	2.0	0.0	16.0		3.2 ± 3.6	2.0	0.0	14.0		.845
GUEF	9.3 ± 5.3	8.0	2.0	23.0		5.8 ± 4.9	4.0	0.0	20.0		.**002**
											
Tinnitus duration (months)	172 ± 106	138	24	400							
THI	21.2 ± 15.3	18.0	2.0	70.0							
Tinnitus burden (self-report; VAS)	3.5 ± 2.4	3.0	1.0	10.0							
Tinnitus ignorability (self-report; VAS)	5.2 ± 2.9	5.0	1.0	10.0							
Tinnitus loudness (self-report; VAS)	5.2 ± 2.5	4.5	2.0	10.0							
Tinnitus loudness (matching, dBA SPL)	52.1 ± 11.2	55.4	24.5	70.5							
Tinnitus frequency (matching, kHz)	5.0 ± 2.2	4.7	0.2	11.4							

*Note.* No differences were found between the two groups, except for a higher GÜF score for the tinnitus group. For the tinnitus group tinnitus parameters are listed in the lower part of the table. Tinnitus group *N* = 32, control group *N* = 31. *PTA* *=* *pure-tone average; MoCA* *=* *Montreal Cognitive Assessment; BDI* *=* *Beck's Depression Inventory; GÜF* *=* *Geräuscheüberempfindlichkeits-Fragebogen (hyperacusis questionnaire); THI* *=* *Tinnitus Handicap Inventory; VAS* *=* *visual analog scale; Stdv* *=* *standard deviation, Min* *=* *minimum, Max* *=* *maximum.*

GÜF scores were different between the two groups, the tinnitus group scoring slightly higher ( W = 717.5, p = .002), but according to the classification of Nelting and Finlayson (2004), only three participants from the tinnitus group and two from the CG fell into the “severe” category, most participants being classified as “mild” or “moderate.”

Among the tinnitus participants, 28 reported experiencing pure-tone-like tinnitus, while four described their tinnitus as noise-like with a discernible peak frequency. On average, participants reported their tinnitus as centrally located, with no strong bias toward either the left or right side (five rather rightwards, four rather leftwards). The average duration of tinnitus was 172 months (*SD* = 106), with a mininum of 2 years, clearly representing a population of individuals with chronic tinnitus. The mean THI score was 21.2 (*SD* = 15.3), suggesting rather mild tinnitus-related distress on average: see Table 3 for more details.

### Suprathreshold Auditory Tests

We present the results of group comparisons using ANCOVA models for each auditory test, controlling for age, gender, education, and PTA. Table 4 displays the statistical values for these models, with the dependent variable in the first column. There were no differences between groups in the PTA ${}_{noise}$ , GDT, AM, FM, DLI, FPT, and DPT tasks. However, there was a trend in one subtest of the AM task (with a modulation frequency of 8 Hz) for participants with tinnitus to perform slightly worse than the CG. When the three subtests were combined this trend disappeared, F (1,57) = 3.8, p = .056, $p_{bonf}$ = .8, $\eta^{2}$ = 0.06. Apart from that, the biggest difference in the suprathreshold auditory variables was for the modulation frequency of 4 Hz in the AM task, F (1,57) = 5.4, p = .024, $p_{bonf}$ = .332, $\eta^{2}$ = 0.09. In the model for PTA ${}_{whitenoise}$ , both PTA, F (1,57) = 79.7, p< .001, $p_{bonf}<$ .001, $\eta^{2}$ = 0.58, and age, F (1,57) = 10.5, p = .002, $p_{bonf}$ = .029, $\eta^{2}$ = 0.15) were influencing factors.

**Table 4.** ANCOVA Results for the Auditory Tests, Including Suprathreshold Auditory Perception and Speech Recognition Tasks.

	Tinnitus group					Control group									
	*M* ± Stdv	*Mdn*	Min	Max		*M* ± Stdv	*Mdn*	Min	Max		MD	*F*-value	*p*	*p*_bonf_	η^2^
PTA_noise_	36.6 ± 3.5	37.2	29.3	44.0		36.2 ± 4.3	35.4	29.3	45.8		0.4	0.99	.323	.999	0.02
GDT	6.4 ± 1.9	6.3	3.0	9.8		6.6 ± 1.4	6.6	3.3	9.8		0.2	0.40	.573	.999	0.01
AM 2Hz	6.6 ± 2.9	5.7	2.3	14.0		7.9 ± 8	5.7	1.9	45.0		1.3	0.04	.841	.999	<0.01
AM 4Hz	5.6 ± 3.4	4.6	1.8	17.3		4.1 ± 2.5	3.5	1.2	14.0		1.5	5.40	.024	.332	0.09
AM 8Hz	5.9 ± 3.3	5.7	2.8	18.2		4.0 ± 1.8	3.5	1.0	9.1		1.9	8.39	.005	.075	0.13
FM 2Hz	0.5 ± 0.3	0.4	0.2	1.4		0.4 ± 0.4	0.4	0.1	1.8		0.1	2.39	.128	.999	0.04
FM 4Hz	0.5 ± 0.2	0.4	0.1	1.4		0.4 ± 0.4	0.4	0.1	1.8		0.1	3.80	.056	.785	0.06
FM 8Hz	0.5 ± 0.2	0.4	0.2	1.1		0.5 ± 0.4	0.4	0.1	1.7		0.0	0.30	.587	.999	0.01
DLI	1.3 ± 0.7	1.1	0.4	2.8		1.4 ± 0.9	1.1	0.4	4.1		0.1	0.01	.943	.999	<0.01
DPT	95.1 ± 7.0	100.0	73.3	100.0		95.6 ± 6.6	100.0	73.3	100.0		0.5	0.00	.213	.999	<0.01
FPT	93.2 ± 8.4	93.3	73.3	100.0		93.2 ± 6.8	93.3	70.0	100.0		0.0	1.46	.233	.999	0.02
SRT	49.1 ± 7.1	48.6	38.0	65.0		45.8 ± 6.1	45.0	35.0	60.0		3.3	0.87	.355	.999	0.02
SiN SNR	0.6 ± 2.7	0.5	−5.0	9.3		−1.9 ± 2.4	−2.5	−5.6	2.5		2.5	11.88	**<**.**001**	.**015**	0.17
GS	55.3 ± 7.2	55.0	43.3	70.0		49.2 ± 7.1	46.7	40.0	68.2		6.1	9.14	.**004**	.**052**	0.14

*Note.* ANCOVA models were controlled for age, gender, education, and PTA. The tinnitus group performed more poorly than the control group for both SiN SNR, F (1,57) = 11.9, p = .001, $p_{bonf}$ = .015, $\eta^{2}$ = 0.17, and GS tasks, F (1,57) = 9.1, p = .004, $p_{bonf}$ = .052, $\eta^{2}$ = 0.14. However, for the GS task the Bonferroni-corrected *p*-value was not significant. PTA = pure-tone average; GDT = gap detection task; AM = amplitude modulation; FM = frequency modulation; DLI = different limen for intensity; DPT = duration pattern test; FPT = frequency pattern test; SRT = speech recognition threshold; SiN SNR = speech-in-noise signal-to-noise ratio; GS = gated speech; Stdv = standard deviation; Min = minimum; Max = maximum; MD = mean difference; *F*  = *F*-value; $p_{bonf}$ = p -value Bonferroni-corrected.

### Speech Auditory Tests

**Figure 2.**
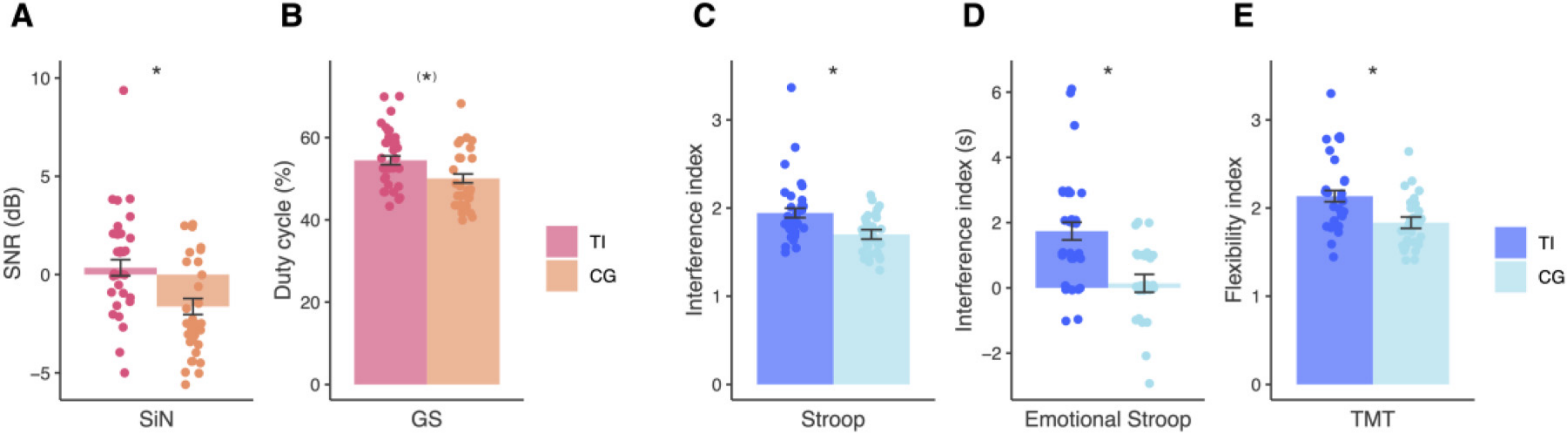
Comparison of speech recognition and cognitive task performance between individuals with and without tinnitus. The bar plots display means and standard errors, with the data corrected for age, gender, education and PTA. (A) SiN SNR in dB. The tinnitus group performed more poorly to the control group with a mean difference of about 2.5 dB, F (1,57) = 11.9, p = .001, $p_{bonf}$ = .015, $\eta^{2}$ = 0.17. (B) GS duty cycle In %. The tinnitus group performed more poorly with a difference of about 6.1%, F (1,57) = 9.14, p = .004, $p_{bonf}$ = .052, $\eta^{2}$ = 0.14. (C) Stroop interference index. Inferior performance of the tinnitus group by 0.3, F (1,57) = 11.9, p = .001, $p_{bonf}$ = .012, $\eta^{2}$ = 0.17. (D) Emotional Stroop interference in seconds. The tinnitus group performed poorer by about 1.5 s, F (1,57) = 16.31, p = 0, $p_{bonf}$ = .002, $\eta^{2}$ = .23). (E) TMT cognitive flexibility index. The tinnitus group performed more poorly than the control group with a mean difference of about 0.3, F (1,57) = 11.4, p = .001, $p_{bonf}$ = .018, $\eta^{2}$ = 0.17. SiN SNR = speech-in-noise signal-to-noise ratio; GS = gated speech; TMT = trail making test; TI = tinnitus group; CG = control group. Asterisks in the figure indicate statistical significance. * p < .05, ^(^*^)^ p *<* *.*10 trend level).

The results of the ANCOVA models for the three speech tests (SRT, SiN, and GS) are displayed in Table 4. The variables that differed between the two groups are presented in Figure 2. For the SRT, we found no differences between the groups, although PTA influenced performance, F (1,57) = 68.7, p< .001, $p_{bonf}<$ .001, $\eta^{2}$ = 0.55. However, for both the SiN and GS tasks, the tinnitus group performed more poorly, with large effect sizes. PTA also influenced performance for both the SiN task, F (1,57) = 18.0, p< .001, $p_{bonf}<$ .001, $\eta^{2}$ = 0.27, and the GS task, F (1,57) = 27.3, p< .001, $p_{bonf}<$ .001, $\eta^{2}$ = 0.32.

**Figure 3.**
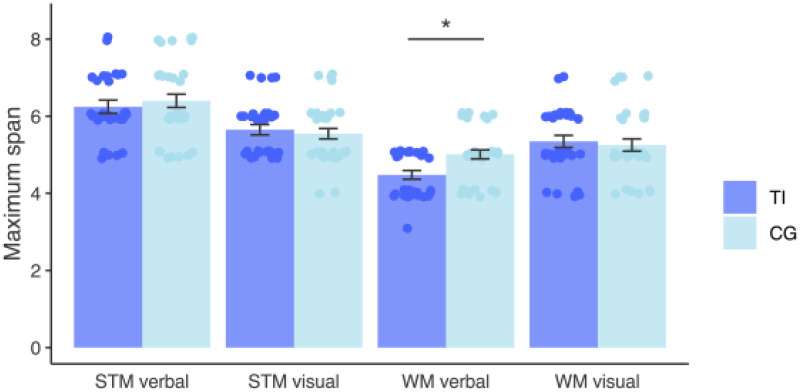
Comparison of short-term and working memory performance between individuals with and without tinnitus. The bar plots display means and standard errors. Verbal working memory performance differed between the two groups, the tinnitus group showing poorer performance with a difference of about 1.4, *F*(1,57) = 9.59, *p* = .003, *p*_*b* *o* *n* *f*_ = .042, *η*^2^ = 0.14. STM = short-term memory; WM = working memory; TI = tinnitus group; CG = control group. Asterisks in the figure indicate statistical significance: * *p* < .05, (*) *p* < .10 (trend level).

SiN: Model 1.1: F (1,57) = 10.3, p = .002, $p_{bonf}$ = .030, $\eta^{2}$ = 0.15; Model 1.2: F (1,57) = 12.3, p = .001, $p_{bonf}$ = .013, $\eta^{2}$ = 0.18.

GS: Model 1.1: F (1,57) = 6.7, p = .012, $p_{bonf}$ = .173, $\eta^{2}$ = 0.10; Model 1.2: F (1,57) = 8.4, p = .006, $p_{bonf}$ = .089, $\eta^{2}$ = 0.12.

### Cognitive Assessment

The results of the ANCOVA models for all cognitive tests are presented in Table 5. The variables that differed between the two groups are shown in Figures 2 and 3. We found group differences in the Stroop interference index, Emotional Stroop interference, the flexibility index, and verbal working memory, the tinnitus group performing more poorly for all these measures, with large effect sizes.

**Table 5.** ANCOVA Results for the Cognitive Tests.

	Tinnitus group					Control group									
	*M* ± Stdv	*Mdn*	Min	Max		*M* ± Stdv	*Mdn*	Min	Max		MD	*F*-value	*p*-value	*p*-value_bonf_	η^2^
Stroop A (s)	12.3 ± 2.2	12.0	9.0	17.0		13.1 ± 2.3	12.0	10.0	19.0		0.8	1.76	.189	.999	0.03
Stroop index	2.0 ± 0.4	1.9	1.5	3.4		1.7 ± 0.2	1.7	1.3	2.2		0.3	11.89	.001	.015	0.17
Emotional Stroop	1.7 ± 1.7	1.0	−1.0	6.0		0.2 ± 1.1	0.0	−3.0	2.0		1.5	16.31	<.001	.002	0.23
TMT A (s)	33.9 ± 10.7	30.0	20.0	57.0		39.3 ± 12.3	39.0	23.0	63.0		5.4	4.62	.036	.506	0.08
TMT index	2.1 ± 0.4	2.0	1.5	3.3		1.8 ± 0.3	1.8	1.4	2.7		0.3	11.44	.001	.018	0.17
Verbal STM (span)	6.2 ± 0.8	6.0	5.0	8.0		6.4 ± 1.0	6.0	5.0	8.0		0.2	0.24	.625	.999	<0.01
Visuell STM (span)	5.7 ± 0.7	6.0	5.0	7.0		5.5 ± 0.8	5.0	4.0	7.0		0.2	0.32	.577	.999	0.01
Verbal WM (span)	4.4 ± 0.6	4.0	3.0	5.0		5.0 ± 0.8	5.0	4.0	6.0		1.4	9.59	.003	.042	0.14
Visuell WM (span)	5.3 ± 0.8	5.0	4.0	7.0		5.3 ± 0.9	5.0	4.0	7.0		0.1	0.39	.533	.999	0.01
PF (unique words)	23.2 ± 7.1	24.0	10.0	38.0		23.8 ± 5.0	23.0	15.0	34.0		0.6	0.40	.527	.999	0.01
DF (unique designs)	36.3 ± 8.2	35.5	21.0	58.0		33.4 ± 7.9	32.0	20.0	50.0		2.9	3.28	.075	.999	0.05
d2-R	146 ± 29	142	97	217		158 ± 29	161	96	221		13	1.43	.237	.999	0.02
DA auditory (ms)	622 ± 111	627	420	868		630 ± 104	639	459	834		7	0.03	.873	.999	<0.01
DA visual (ms)	837 ± 127	803	681	1286		893 ± 128	848	754	1330		55	4.47	.039	.544	0.07

*Note.* ANCOVA models were controlled for age, gender, education, and PTA. The tinnitus group had a lower stroop interference index, F (1,57) = 11.89, p = .001, $p_{bonf}$ = .012, $\eta^{2}$ = 0.17, poorer Emotional Stroop interference, F (1,57) = 16.31, p < .001, $p_{bonf}$ = .002, $\eta^{2}$ = 0.23, a reduced TMT flexibility index, F (1,57) = 11.44, p = .001, $p_{bonf}$ = .018, $\eta^{2}$ = 0.17, and a lower verbal working memory score, F (1,57) = 9.59, p = .003, $p_{bonf}$ = .042, $\eta^{2}$ = 0.14) compared to the control group. TMT = trail making test; STM = short-term memory; WM = working memory; PF = phonemic fluency; DF = design fluency; DA = divided attention; Stdv = standard deviation; Min = minimum; Max = maximum; MD = mean difference; $p_{bonf}$ = p -value Bonferroni-corrected.

For the Stroop A test (processing speed), age had a negative effect on performance, F (1,57) = 10.7, p = .001, $p_{bonf}$ = .008, $\eta^{2}$ = 0.17.

For the modality comparison, we used ANCOVA models with an interaction term between group and task type. These analyses included comparisons for working memory task type (verbal vs. visual), fluency (phonemic vs. design), and divided attention (auditory vs. visual). For the working memory task, there was an effect of task type, F (1,61) = 24.9, p< .001, $\eta^{2}$ = 0.29, likely reflecting general modality differences. Additionally, there was a significant interaction between task type and group, F (1,61) = 9.1, p = .004, $\eta^{2}$ = 0.13, indicating that individuals with tinnitus performed more poorly than the CG only in the verbal working memory task. For fluency, there was an effect of fluency type, likely due to inherent task differences, F (1,61) = 109.3, p< .001, $\eta^{2}$ = 0.64. However, there was no interaction between fluency type and group, F (1,61) = 2.8, p = .100, $\eta^{2}$ = 0.04, suggesting that performance patterns across fluency types were similar for the two groups. In the divided attention task, there was an effect of modality, F (1,61) = 220.8, p< .001, $\eta^{2}$ = 0.78, with faster reaction times for the auditory modality. There was no interaction between modality and group, F (1,61) = 1.1, p = .303, $\eta^{2}$ = 0.02, indicating that performance patterns across modalities were similar for the two groups.

### Secondary Analyses

**Figure 4.**
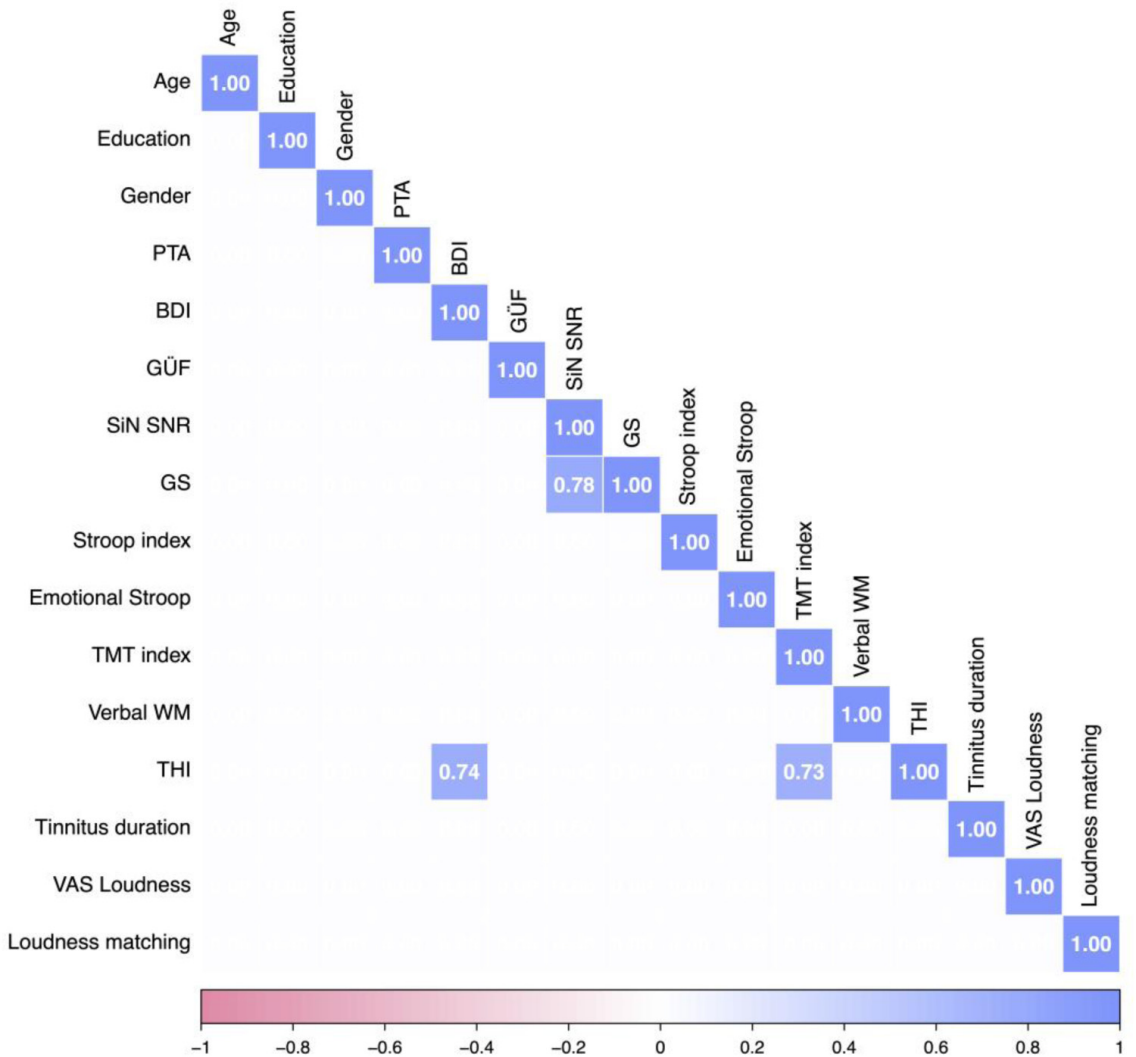
Spearman bivariate correlation matrix of variables for individuals with tinnitus. Only correlations with Bonferroni corrected p-values smaller than .05 are displayed. Expected positive correlations between SiN and GS, as well as between BDI and THI. Further, there is a positive correlation between THI and the TMT index. PTA = pure-tone average; BDI = Beck's Depression Inventory; GÜF = Geräuscheüberempfindlichkeits-Fragebogen; SiN SNR = speech-in-noise signal-to-noise ratio; GS = gated speech; STM = short-term memory; WM = working memory; THI = Tinnitus Handicap Inventory; VAS = visual analog scale.

We computed a correlation matrix of scores for the tinnitus group to examine potential relationships, focusing on the correlations between the THI scores and self-reported tinnitus loudness (VAS) and other variables, see Figure 4 and Supplemental Figure S3 (for uncorrected correlation matrix).

The correlation matrix revealed several expected associations, including significant correlations between SiN performance and GS performance, between THI scores and BDI scores, and between THI scores and the TMT flexibility index. Based on these correlations and in line with previous research, we constructed ANCOVA models to predict cognitive performance (Stroop, TMT) as well as SiN performance from THI scores, while controlling for age, gender, education, and PTA.

**Figure 5.**
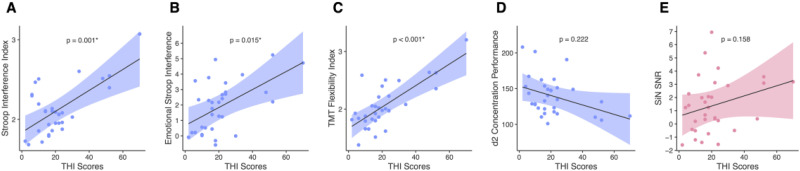
ANCOVA models of the relationship between cognitive variables, SiN performance, and tinnitus variables. The plots display the ANCOVA models adjusted for age, gender, education, and PTA. The p-values are added to each plot. (A) Shows a positive borrelation between the Stroop interference index and THI scores. (B) Indicates a positive correlation between the emotional Stroop interference (measured in seconds) and THI scores. (C) Shows a positive correlation between the TMT flexibility index and the THI. (D) Shows the association between d2 concentration performance and THI scores. (E) Shows the association between the SiN SNR and THI scores. THI = Tinnitus Handicap Inventory; TMT = trail making test; SiN SNR = speech-in-noise signal-to-noise ratio.

The analysis revealed associations between THI scores and the Stroop interference index ( β = 0.01, p< .001, $p_{bonf}$ = .001), the Emotional Stroop index ( β = 0.06, *p*  = 0.004, $p_{bonf}$ = .015), and the TMT index ( β = 0.02, p< .001, $p_{bonf}<$ .001), see plots in Figure 5. We further included d2 performance, which showed a trend-level association with THI scores ( β = −0.7, p< .055, $p_{bonf}<$ .222). In each of these models, higher THI scores were associated with poorer performance.

We also included SiN performance which showed no association with THI scores ( β = 0.0, p = .158).

To examine if tinnitus effects were simply additive or whether they varied with age or hearing loss severity, we included interaction terms between group (tinnitus vs. control) and age, and between group and PTA, in each model. In the auditory tests (SiN and GS), no significant Group × Age (SiN: F (1, 56) = 0.7, p = .398; GS: F (1, 56) = 0.3, p = .611) or Group × PTA interaction was observed (SiN: F (1, 56) = 0.0, p = .879; GS: F (1, 56) = 0.1, p = .711). Similarly, no significant interactions between age and group were found for the cognitive measures (Stroop: F (1, 56) = 0.1, p = 0.715; Emotional Stroop: F (1, 56) = 0.5, p = .470; TMT: F (1, 56) = 0.9, p = .339; and verbal working memory: F (1, 56) = 3.0, p = .088).

## Discussion

Our results indicate poorer speech recognition in challenging listening environments (SiN and GS tasks), as well as poorer EFs (interference control, emotional interference, cognitive flexibility, and verbal working memory) for individuals with tinnitus compared to controls. Exploratory analyses showed correlations between several of the cognitive variables and the extent of tinnitus distress within the tinnitus group. These results align with previous studies on younger populations, suggesting that speech recognition difficulties in challenging listening environments as well as EF deficits occur for individuals with tinnitus across different age groups and may even be more pronounced in older age.

### Tinnitus and Suprathreshold Auditory Perception

Our investigation of suprathreshold auditory perception revealed no differences between the tinnitus group and CG across a comprehensive battery of threshold and suprathreshold tests (PTA ${}_{noise}$ , GDT, AM, FM, DLI, FPT, and DPT). These findings align with our initial hypotheses and corroborate previous research in the field (e.g., Boyen et al., 2015; Bureš et al., 2019; Gilani et al., 2013; Raj-Koziak et al., 2022; Zeng et al., 2020). It is important, however, to acknowledge that our null findings here contrast with some prior literature that has reported suprathreshold auditory deficits in tinnitus (Fournier & Hébert, 2013; Ibraheem & Hassaan, 2017; e.g., Paul et al., 2017; Weisz et al., 2006). Several factors could account for this discrepancy. One key reason may be the relatively low tinnitus distress in our sample, as more severe symptoms are often associated with more pronounced deficits. Furthermore, the sensitivity of the specific tasks used could be a factor, which may not have been sufficiently challenging to unmask subtle processing inefficiencies. Finally, it is plausible that older adults, particularly those with long-standing tinnitus, may develop neural or cognitive compensatory mechanisms that allow them to maintain normal performance on these structured laboratory tasks, even if underlying deficits exist. Taken together, given the similar performance of the two groups in suprathreshold auditory tasks, it is reasonable to expect that the observed differences in speech recognition tasks are not due to differences in suprathreshold auditory perception in our study.

### Tinnitus and Speech Perception

Our analyses demonstrated that older adults with tinnitus exhibit inferior speech recognition in challenging listening environments (SiN and GS tasks). Despite the influence of hearing loss on SiN performance, tinnitus may introduce an additional difficulty on top of that. These findings align with our previous study (Sommerhalder et al., 2023) as well as with the majority of other previous studies, primarily conducted on (younger) adults (e.g., Gilles et al., 2016; Liu et al., 2020; Niewiarowicz et al., 2022; Vielsmeier et al., 2016). The difference between the two groups of our former study with younger participants was smaller than in the current study. This hints at more pronounced difficulties in older adults with tinnitus, compared to (younger) adults.

There are barely any studies investigating SiN performance for older adults with tinnitus and those that exist have yielded mixed results. For instance, Oosterloo et al. (2020) demonstrated poorer SiN performance for tinnitus patients, but only among those who also experienced hearing loss. In contrast, Bureš et al. (2019) found no differences between the tinnitus and CGs. However, their data showed a nonsignificant trend in the same direction as our results, with the tinnitus group performing slightly worse than controls.

Due to the limited research available, drawing (definitive) conclusions is challenging. However, our results extend current research by demonstrating that SiN deficits affect older adults with tinnitus similarly to, or even more strongly than, for younger adults.

The GS task assesses speech recognition of periodically gated speech. For this task the tinnitus group performed more poorly than the CG. Although this difference failed to survive Bonferroni correction, the effect size was substantial, suggesting a meaningful difference in GS performance between the two groups. This finding indicates that not only SiN recognition but also other forms of challenging speech recognition are affected in older adults with tinnitus, and aligns with our previous study.

The GS task requires participants to actively integrate and interpret fragmented speech stimuli. This process engages higher-level control mechanisms, temporal processing abilities, contextual aspects, and various cognitive factors (Moradi et al., 2014a, 2014b). Individuals with tinnitus may exhibit altered central neural mechanisms that hinder their ability to effectively utilize contextual cues and suppress irrelevant information during SiN and GS recognition tasks. This impairment likely results in poorer performance not only in experimental tasks but also in day-to-day situations.

To our knowledge, only three prior studies have investigated GS task performance for individuals with tinnitus (Bureš et al., 2019, 2024; Sommerhalder et al., 2023). Bureš et al. (2019) also investigated older adults and found comparable performance of tinnitus and CGs. This discrepancy may be attributed to differences in sample characteristics. While the participants in their study were of similar age to those in our sample, they had better hearing overall, with lower pure-tone averages.

Since GS performance is affected by hearing loss (Fogerty et al., 2022; Profant et al., 2020), this could play a role. Additionally, variations in speech processing due to maternal language differences between the Czech and Swiss German populations, as well as differences in sentence structure (particularly contextual aspects), may have influenced the results.

To summarize, we confirmed our hypothesis regarding speech recognition, demonstrating that older adults with tinnitus also experience speech recognition deficits in challenging listening environments. These deficits appear to be more pronounced than those observed for younger adults. Furthermore, these deficits extend beyond SiN performance, manifesting in another test of challenging speech recognition.

### Tinnitus and Cognition

Our results demonstrated inferior performance of older individuals with tinnitus in EFs (in the Stroop, the Emotional Stroop, the TMT, and the backwards digit span tasks). Individuals with tinnitus exhibited poorer interference control in the Stroop task compared to the CG, while visuoverbal processing speed was similar across groups. These findings are consistent with our hypothesis and are supported by previous behavioral and neuroimaging studies (Araneda et al., 2015, 2018; Gonendik et al., 2021; Stevens et al., 2007). Our results suggest that older adults with tinnitus experience difficulties with interference control. Araneda et al. (2015) interpreted this poorer interference control as a sign of altered central executive control in individuals with tinnitus.

The results of the Emotional Stroop task indicate increased interference among older individuals with tinnitus. This suggests that tinnitus-related words have a stronger impact on cognitive processing in individuals with tinnitus compared to controls. Tinnitus may affect not only general EFs but also emotional processing/emotional cognitive control. However, previous studies with tinnitus individuals have not demonstrated a clear Emotional Stroop effect, which may be due to differences in task and samples (Andersson et al., 2000; Golm et al., 2016).

The TMT results indicate reduced cognitive flexibility in older individuals with tinnitus, as evidenced by poorer performance in the TMT flexibility index. This is in line with our hypothesis and corroborates existing behavioral studies (Chen et al., 2018; Gabr et al., 2011; Pajor et al., 2013; Stevens et al., 2007; Vanneste et al., 2016). This cognitive flexibility deficit may indicate that individuals with tinnitus experience challenges in task-switching and juggling with concurrent cognitive demands. We found comparable processing speeds for the TMT-A for the tinnitus and CGs, which contrasts with some previous studies (Chen et al., 2018; Gabr et al., 2011). Differences in the characteristics of the participants, such as age, severity of tinnitus, cognitive reserve, or comorbid conditions, might have influenced the results.

We found poorer verbal working memory for individuals with tinnitus than controls, while verbal short-term memory did not differ across groups. This finding is consistent with existing literature (Nagaraj et al., 2020; Rossiter et al., 2006; Trevis et al., 2016). Additionally, we found that the impairment is specific to the verbal modality and did not differ for visual–spatial working memory. This selective impairment of verbal working memory may indicate that the persistent internal sound occupies cognitive resources, potentially hindering the efficient storage and rehearsal of verbal information. In contrast, short-term memory performance appeared unaffected, suggesting that basic storage capacity is intact, while more complex processes involving manipulation and central executive control could be more vulnerable (Baddeley, 2000; Donolato et al., 2017).

In contrast, some EF measures showed no group differences. Contrary to expectations, tinnitus and CGs performed similarly on phonemic verbal fluency, diverging from reports of reduced semantic fluency in tinnitus (Cardon et al., 2019; Hallam et al., 2004). This may reflect task type, as phonemic fluency relies more on lexical retrieval than semantic memory (Birn et al., 2010; Henry & Crawford, 2004). Design fluency also did not differ between groups.

Similarly, no group differences emerged on the d2 test, consistent with some studies that show deficits in concentration (Hallam et al., 2004; Stevens et al., 2007) but not others (McKenna et al., 1995; McKenna & Hallam, 1999). Since d2 norms extend only to age 60, the task may have been overly demanding for older participants, increasing variability and masking group effects. Its strong visual demands may also have enabled compensatory strategies, reducing sensitivity to subtle deficits.

Divided attention performance showed no group differences across modalities or outcomes, contrasting with earlier findings (Hallam et al., 2004; Rossiter et al., 2006). The simplicity of our paradigm and the structured laboratory setting may have limited ecological validity and sensitivity to small effects.

Across all tasks, the relatively low tinnitus distress in our sample (THI scores) offers another likely explanation, as higher distress is linked to stronger attentional disruption (Brueggemann et al., 2021), see also Figure 5. Individuals with low-distress tinnitus may recruit compensatory resources to maintain performance.

Overall, we could confirm some of our hypotheses regarding EFs as our results reveal reduced executive cognitive control for older individuals with tinnitus, as evidenced by poorer performance in interference control, emotional interference, cognitive flexibility, and verbal working memory tasks but we could not confirm our hypotheses regarding phonemic fluency and attentional functions (concentration performance and divided attention).

#### Secondary Analyses

Our analyses within the tinnitus group revealed correlations between several EF measures and tinnitus distress. Greater tinnitus distress is associated with poorer EFs. Such results align with the hypothesis that tinnitus acts as a chronic stressor that persistently engages attentional and executive resources to manage its associated distress and annoyance, thereby diminishing cognitive capacity for other tasks and disrupting cognitive control processes (Andersson & McKenna, 2006; Khan & Husain, 2020).

### Altered Central Processes as Underlying Factors in SiN Recognition and EF Deficits

Our findings favor the hypothesis that altered central mechanisms, which encompass auditory processes, cognitive functions, and/or a combination of the two, may underlie the deficits in SiN perception, GS recognition, and EFs observed for individuals with chronic subjective tinnitus. There were no differences in SRT or suprathreshold auditory perception between the two groups. This strengthens the idea that impaired speech recognition in challenging listening environments for individuals with tinnitus is associated with deficient central mechanisms rather than issues with peripheral auditory function. Our results corroborate the hypothesis proposed by Tai and Husain (2019), that tinnitus disrupts cognitive control processing. Additionally, the correlation between EF scores and tinnitus distress supports the hypothesis that tinnitus disrupts cognitive resources, thereby reducing cognitive capacity for other tasks (Andersson & McKenna, 2006; Khan & Husain, 2020).

Additional exploratory analyses revealed that there were no interactions between group and age as well as group and PTA. These findings indicate that the tinnitus-related differences in auditory and cognitive performance were stable across the observed range of ages and hearing thresholds. In other words, the effect of tinnitus did not depend on whether participants were younger or older within our sample, nor on the severity of their hearing loss. This pattern suggests that tinnitus contributes an independent burden beyond that expected from age-related decline or hearing loss alone, and that the observed deficits cannot be explained by disproportionate age-related decline or greater hearing loss severity in the tinnitus group.

### Limitations

Several limitations of this study warrant consideration. Firstly, the CG had better hearing thresholds than the tinnitus group. Differences in hearing ability could influence the outcomes independently of the presence or absence of tinnitus. To account for this aspect we included and controlled for hearing thresholds in all our models and further calculated Models 1.1 and 1.2. It should also be noted that higher frequencies—including those above 8 kHz—contribute to SiN performance (Polspoel et al., 2022; Trine & Monson, 2020), which were not assessed in the present study. Such extended high-frequency hearing loss may also contribute to deficits in individuals with tinnitus. Recent evidence suggests that tinnitus can be associated with degraded extended high-frequency thresholds even in those with normal audiograms at conventional frequencies (Jafari et al., 2022; Song et al., 2021).

Secondly, tinnitus severity/distress might have influenced the results of our study. THI scores were relatively low for our sample, falling predominantly within the “mild” category.

Third, psychoacoustic measurements are inherently subjective, as they depend on each participant's individual decision thresholds. Similarly, examiner judgment can introduce subjectivity into the assessment process. In our study, six auditory tests were fully automated, while five required examiner judgment, which could have introduced variability. To minimize this risk and ensure consistency, all examiner-dependent tests were conducted by the same investigator following a standardized measurement protocol.

The cross-sectional nature of our study design prevents us from drawing conclusions about causality or the progression of the observed associations over time. Specifically tailored longitudinal research is warranted to address this shortcoming. Furthermore, a related limitation is the absence of mechanistic or neurophysiological evidence to substantiate our claims. While our behavioral results suggest central auditory involvement, we did not collect direct physiological data (e.g., EEG or fMRI) to support this interpretation.

The tinnitus group had slightly higher levels of hyperacusis than the CG. However, according to the classification of Nelting (2003), most participants in both the tinnitus and CGs had “mild” or “moderate” hyperacusis. Only a small subset of participants from both groups met the criteria for “severe” hyperacusis. Due to the subjective nature of questionnaires, it would be beneficial to also evaluate hyperacusis behaviorally.

Another limitation is that the auditory stimuli were not tailored to match each participant's tinnitus characteristics (e.g., perceived frequency or loudness). Using stimuli that correspond more closely to the individual tinnitus percept might have influenced task performance and potentially revealed different patterns between the tinnitus and CGs.

Another important aspect to consider is task difficulty, particularly in the cognitive tests. However, since most of the neuropsychological tests employed in this study are standardized and age-normed, it is unlikely that difficulty alone explains the observed group differences.

Looking at the population studied, the culturally and linguistically homogeneous sample of Swiss German speakers may limit the generalizability of the results to other populations. Lastly, our study did not incorporate an assessment of personality traits. This is important given the emerging evidence linking personality characteristics to both tinnitus (Langguth et al., 2007b; Simões et al., 2019) and speech-processing abilities (Wöstmann et al., 2021).

### Future Research

Future research in the field of tinnitus should address several critical areas highlighted by our findings. To advance, it is imperative to conduct more comprehensive behavioral studies of older adults involving extensive psychometric data collection.

Longitudinal studies are needed to explore the relationship between tinnitus and central mechanisms over time. This approach may help clarify whether tinnitus disrupts cognitive processes or if preexisting disruptions in cognitive processes serve as a risk factor for developing tinnitus.

Regarding speech recognition, we recommend incorporating both highly controlled and naturalistic stimuli. While controlled stimuli allow for precise manipulation of specific variables, naturalistic stimuli offer ecological validity and better reflect real-world listening situations. To achieve this in future work, we are currently building a 3D audio laboratory. This facility will enable the simulation of realistic acoustic environments with precise spatial control, allowing us to present speech embedded in complex, lifelike soundscapes while still manipulating experimental parameters in a controlled manner.

To gain deeper insights into central auditory processing for individuals with tinnitus, future research should collect and analyze neurophysiological data. Advanced neuroimaging and electrophysiological techniques can reveal underlying neural mechanisms and potential compensatory strategies employed by individuals with tinnitus. Exploring potential changes over time using these methods represents a promising approach, especially in studies that follow individuals from the onset of tinnitus. Such studies may help identify critical periods for intervention and inform the development of targeted treatment strategies. Future studies could explore multimodal intervention approaches, combining auditory training with cognitive rehabilitation techniques. Given the dual involvement of perceptual and executive domains observed in individuals with tinnitus, integrated interventions may prove more effective than isolated treatments. Examining the efficacy and timing of such combined approaches could be a productive direction for both clinical and translational research.

Finally, standardization of assessment protocols encompassing both auditory and cognitive measures would facilitate comparisons across studies, or even multicenter studies, and enable more robust meta-analyses in the future.

## Conclusion

In conclusion, our study extends existing research on speech recognition and cognition for individuals with tinnitus by comprehensive testing of speech-related and cognitive mechanisms, specifically targeting older adults. Our results demonstrate deficits in challenging speech recognition processes (SiN and GS tasks) that seem to be more pronounced for older adults than for younger adults with tinnitus. Suprathreshold auditory perception, however, was comparable for the two groups. We identified specific deficits in EF among tinnitus participants (in interference control, emotional interference, cognitive flexibility, and verbal working memory). Performance of the EF tasks was negatively related to tinnitus distress. The causal relationships and potential interactions between EF and speech recognition remain to be fully resolved.

Altogether, our findings underscore the far-reaching effects of tinnitus beyond auditory perception, extending into higher-order cognitive processes/central mechanisms, particularly in older adults with tinnitus. This underscores the real-life challenges faced by older individuals with tinnitus, highlighting the burden due to impaired communication and cognitive difficulties. Our study corroborates the everyday experiences reported by individuals with tinnitus and supports the hypothesis of altered central mechanisms.

## Supplemental Material

sj-docx-1-tia-10.1177_23312165251389585 - Supplemental material for Association of Tinnitus With Speech Recognition and Executive Functions in Older Adults Supplemental material, sj-docx-1-tia-10.1177_23312165251389585 for Association of Tinnitus With Speech Recognition and Executive Functions in Older Adults by Nick Sommerhalder, Zbyněk Bureš, Oliver Profant, Tobias Kleinjung, Patrick Neff and Martin Meyer in Trends in Hearing
