# Supplementary material for: Association of Tinnitus With Speech Recognition and Executive Functions in Older Adults
Source: Trends Hear. 2025 Nov 13;29:23312165251389585. doi: 10.1177/23312165251389585 (PMC12615926; doi:10.1177/23312165251389585)
Supplement: sj-docx-1-tia-10.1177_23312165251389585 - Supplemental material for Association of Tinnitus With Speech Recognition and Executive Functions in Older Adults [file sj-docx-1-tia-10.1177_23312165251389585.docx]

**Supplementary Materials**

Supplementary Figure 1. **Spearman bivariate correlation matrix for individuals with tinnitus.** Only correlations with Bonferroni corrected *p*-values smaller than 0.05 are displayed. *PTA = puretone average, BDI = Beck’s Depression Inventory, GÜF = GeräuscheüberempfindlichkeitsFragebogen, GDT = gap detection task, AM = amplitude modulation, FM = frequency modulation, DLI = different limen for intensity, DPT = duration pattern test, FPT = frequency pattern test, SRT = speech recognition threshold, SiN SNR = speech-in-noise signal-to-noise ratio, GS = gated speech, STM = short-term memory, WM = working memory, PF = phonemic fluency, DF = design fluency, DA = divided attention, THI = Tinnitus Handicap Inventory, VAS = visual analog scale.*

| **1.00** |  | | | | | | | | | | | | | | | | | | | | | | | | | | | | | | | | |
| --- | --- | --- | --- | --- | --- | --- | --- | --- | --- | --- | --- | --- | --- | --- | --- | --- | --- | --- | --- | --- | --- | --- | --- | --- | --- | --- | --- | --- | --- | --- | --- | --- | --- |
|  |  | | | | | | | | | | | | | | | | | | | | | | | | | | | | | | | | |
| **0.00** | **1.00** | Gender | | | | | | | | | | | | | | | | | | | | | | | | | | | | | | | |
| **0.00** | **0.00** | **1.00** | PTA | | | | | | | | | | | | | | | | | | | | | | | | | | | | | | |
| **0.00** | **0.00** | **0.00** | **1.00** | BDI | | | | | | | | | | | | | | | | | | | | | | | | | | | | | |
| **0.00** | **0.00** | **0.00** | **0.00** | **1.00** | GÜF  PTAnoise | | | | | | | | | | | | | | | | | | | | | | | | | | | | |
| **0.00** | **0.00** | **0.00** | **0.00** | **0.00** | **1.00** |  | | | | | | | | | | | | | | | | | | | | | | | | | | | |
| **0.00** | **0.00** | **0.00** | **0.75** | **0.00** | **0.00** | **1.00** | GDT | | | | | | | | | | | | | | | | | | | | | | | | | | |
| **0.00** | **0.00** | **0.00** | **0.00** | **0.00** | **0.00** | **0.00** | **1.00** | AM | | | | | | | | | | | | | | | | | | | | | | | | | |
| **0.00** | **0.00** | **0.00** | **0.00** | **0.00** | **0.00** | **0.00** | **0.00** | **1.00** | FM | | | | | | | | | | | | | | | | | | | | | | | | |
| **0.00** | **0.00** | **0.00** | **0.00** | **0.00** | **0.00** | **0.00** | **0.00** | **0.00** | **1.00** | DLI | | | | | | | | | | | | | | | | | | | | | | | |
| **0.00** | **0.00** | **0.00** | **0.00** | **0.00** | **0.00** | **0.00** | **0.00** | **0.00** | **0.00** | **1.00** | DPT | | | | | | | | | | | | | | | | | | | | | | |
| **0.00** | **0.00** | **0.00** | **0.00** | **0.00** | **0.00** | **0.00** | **0.00** | **0.00** | **0.00** | **0.00** | **1.00** | FPT | | | | | | | | | | | | | | | | | | | | | |
| **0.00** | **0.00** | **0.00** | **0.00** | **0.00** | **0.00** | **0.00** | **0.00** | **0.00** | **0.00** | **0.00** | **0.76** | **1.00** | SRT | | | | | | | | | | | | | | | | | | | | |
| **0.00** | **0.00** | **0.00** | **0.00** | **0.00** | **0.00** | **0.00** | **0.00** | **0.00** | **0.00** | **0.00** | **0.00** | **0.00** | **1.00** | SiN SNR | | | | | | | | | | | | | | | | | | | |
| **0.00** | **0.00** | **0.00** | **0.00** | **0.00** | **0.00** | **0.68** | **0.00** | **0.00** | **0.00** | **0.00** | **0.00** | **0.00** | **0.00** | **1.00** | GS | | | | | | | | | | | | | | | | | | |
| **0.00** | **0.00** | **0.00** | **0.00** | **0.00** | **0.00** | **0.00** | **0.00** | **0.00** | **0.00** | **0.00** | **0.00** | **0.00** | **0.00** | **0.78** | **1.00** | Stroop A  Stroop index | | | | | | | | | | | | | | | | | |
| **0.00** | **0.00** | **0.00** | **0.00** | **0.00** | **0.00** | **0.00** | **0.00** | **0.00** | **0.00** | **0.00** | **0.00** | **0.00** | **0.00** | **0.00** | **0.00** | **1.00** | Emotional Stroop | | | | | | | | | | | | | | | | |
| **0.00** | **0.00** | **0.00** | **0.00** | **0.00** | **0.00** | **0.00** | **0.00** | **0.00** | **0.00** | **0.00** | **0.00** | **0.00** | **0.00** | **0.00** | **0.00** | **0.00** | **1.00** |  | | | | | | | | | | | | | | | |
| **0.00** | **0.00** | **0.00** | **0.00** | **0.00** | **0.00** | **0.00** | **0.00** | **0.00** | **0.00** | **0.00** | **0.00** | **0.00** | **0.00** | **0.00** | **0.00** | **0.00** | **0.00** | **1.00** | TMT A  TMT index | | | | | | | | | | | | | | |
| **0.00** | **0.00** | **0.00** | **0.00** | **0.00** | **0.00** | **0.00** | **0.00** | **0.00** | **0.00** | **0.00** | **0.00** | **0.00** | **0.00** | **0.00** | **0.00** | **0.00** | **0.00** | **0.00** | **1.00** | STM verbal | | | | | | | | | | | | | |
| **0.00** | **0.00** | **0.00** | **0.00** | **0.00** | **0.00** | **0.00** | **0.00** | **0.00** | **0.00** | **0.00** | **0.00** | **0.00** | **0.00** | **0.00** | **0.00** | **0.00** | **0.00** | **0.00** | **0.00** | **1.00** | WM verbal | | | | | | | | | | | | |
| **0.00** | **0.00** | **0.00** | **0.00** | **0.00** | **0.00** | **0.00** | **0.00** | **0.00** | **0.00** | **0.00** | **0.00** | **0.00** | **0.00** | **0.00** | **0.00** | **0.00** | **0.00** | **0.00** | **0.00** | **0.00** | **1.00** | STM visual | | | | | | | | | | | |
| **0.00** | **0.00** | **0.00** | **0.00** | **0.00** | **0.00** | **0.00** | **0.00** | **0.00** | **0.00** | **0.00** | **0.00** | **0.00** | **0.00** | **0.00** | **0.00** | **0.00** | **0.00** | **0.00** | **0.00** | **0.00** | **0.00** | **1.00** | WM visual | | | | | | | | | | |
| **0.00** | **0.00** | **0.00** | **0.00** | **0.00** | **0.00** | **0.00** | **0.00** | **0.00** | **0.00** | **0.00** | **0.00** | **0.00** | **0.00** | **0.00** | **0.00** | **0.00** | **0.00** | **0.00** | **0.00** | **0.00** | **0.00** | **0.00** | **1.00** |  | | | | | | | | | |
| **0.00** | **0.00** | **0.00** | **0.00** | **0.00** | **0.00** | **0.00** | **0.00** | **0.00** | **0.00** | **0.00** | **0.00** | **0.00** | **0.00** | **0.00** | **0.00** | **0.00** | **0.00** | **0.00** | **0.00** | **0.00** | **0.00** | **0.00** | **0.00** | **1.00** | d2 | | | | | | | | |
| **0.00** | **0.00** | **0.00** | **0.00** | **0.00** | **0.00** | **0.00** | **0.00** | **0.00** | **0.00** | **0.00** | **0.00** | **0.00** | **0.00** | **0.00** | **0.00** | **0.00** | **0.00** | **0.00** | **0.00** | **0.00** | **0.00** | **0.00** | **0.00** | **0.00** | **1.00** | PF | | | | | | | |
| **0.00** | **0.00** | **0.00** | **0.00** | **0.00** | **0.00** | **0.00** | **0.00** | **0.00** | **0.00** | **0.00** | **0.00** | **0.00** | **0.00** | **0.00** | **0.00** | **0.00** | **0.00** | **0.00** | **0.00** | **0.00** | **0.00** | **0.00** | **0.00** | **0.00** | **0.00** | **1.00** | DF  DA auditory | | | | | | |
| **0.00** | **0.00** | **0.00** | **0.00** | **0.00** | **0.00** | **0.00** | **0.00** | **0.00** | **0.00** | **0.00** | **0.00** | **0.00** | **0.00** | **0.00** | **0.00** | **0.00** | **0.00** | **0.00** | **0.00** | **0.00** | **0.00** | **0.00** | **0.00** | **0.00** | **0.00** | **0.00** | **1.00** |  | | | | | |
| **0.00** | **0.00** | **0.00** | **0.00** | **0.00** | **0.00** | **0.00** | **0.00** | **0.00** | **0.00** | **0.00** | **0.00** | **0.00** | **0.00** | **0.00** | **0.00** | **0.00** | **0.00** | **0.00** | **0.00** | **0.00** | **0.00** | **0.00** | **0.00** | **0.00** | **0.00** | **0.00** | **0.00** | **1.00** | DA visual | | | | |
| **0.00** | **0.00** | **0.00** | **0.00** | **0.00** | **0.00** | **0.00** | **0.00** | **0.00** | **0.00** | **0.00** | **0.00** | **0.00** | **0.00** | **0.00** | **0.00** | **0.00** | **0.00** | **0.00** | **0.00** | **0.00** | **0.00** | **0.00** | **0.00** | **0.00** | **0.00** | **0.00** | **0.00** | **0.00** | **1.00** | THI  Tinnitus duration | | | |
| **0.00** | **0.00** | **0.00** | **0.00** | **0.74** | **0.00** | **0.00** | **0.00** | **0.00** | **0.00** | **0.00** | **0.00** | **0.00** | **0.00** | **0.00** | **0.00** | **0.00** | **0.00** | **0.00** | **0.00** | **0.73** | **0.00** | **0.00** | **0.00** | **0.00** | **0.00** | **0.00** | **0.00** | **0.00** | **0.00** | **1.00** | VAS Loudness | | |
| **0.00** | **0.00** | **0.00** | **0.00** | **0.00** | **0.00** | **0.00** | **0.00** | **0.00** | **0.00** | **0.00** | **0.00** | **0.00** | **0.00** | **0.00** | **0.00** | **0.00** | **0.00** | **0.00** | **0.00** | **0.00** | **0.00** | **0.00** | **0.00** | **0.00** | **0.00** | **0.00** | **0.00** | **0.00** | **0.00** | **0.00** | **1.00** | Loudness matching | |
| **0.00** | **0.00** | **0.00** | **0.00** | **0.00** | **0.00** | **0.00** | **0.00** | **0.00** | **0.00** | **0.00** | **0.00** | **0.00** | **0.00** | **0.00** | **0.00** | **0.00** | **0.00** | **0.00** | **0.00** | **0.00** | **0.00** | **0.00** | **0.00** | **0.00** | **0.00** | **0.00** | **0.00** | **0.00** | **0.00** | **0.00** | **0.00** | **1.00** |  |
| **0.00** | **0.00** | **0.00** | **0.00** | **0.00** | **0.00** | **0.00** | **0.00** | **0.00** | **0.00** | **0.00** | **0.00** | **0.00** | **0.00** | **0.00** | **0.00** | **0.00** | **0.00** | **0.00** | **0.00** | **0.00** | **0.00** | **0.00** | **0.00** | **0.00** | **0.00** | **0.00** | **0.00** | **0.00** | **0.00** | **0.00** | **0.00** | **0.00** | **1.00** |

Age

Age

Education

Education

Gender

PTA

BDI

GÜF

PTAnoise

GDT

AM

FM

DLI

DPT

FPT

SRT

SiN SNR

GS

Stroop A

Stroop index

Emotional Stroop

TMT A

TMT index

STM verbal

WM verbal

STM visual

WM visual d2 PF

DF

DA auditory

DA visual

THI

Tinnitus duration

VAS Loudness

Loudness matching

−1 −0.8 −0.6 −0.4 −0.2 0 0.2 0.4 0.6 0.8 1

Supplementary Figure 2. **Spearman bivariate correlation matrix for all participants.** Only correlations with Bonferroni corrected *p*-values smaller than 0.05 are displayed. *PTA = pure-tone average, BDI = Beck’s Depression Inventory, GÜF = Geräuscheüberempfindlichkeits-Fragebogen, GDT = gap detection task, AM = amplitude modulation, FM = frequency modulation, DLI = different limen for intensity, DPT = duration pattern test, FPT = frequency pattern test, SRT = speech recognition threshold, SiN SNR = speech-in-noise signal-to-noise ratio, GS = gated speech, STM = short-term memory, WM = working memory, PF = phonemic fluency, DF = design fluency, DA = divided attention.*

| **1.00** |  | | | | | | | | | | | | | | | | | | | | | | | | | | | | |
| --- | --- | --- | --- | --- | --- | --- | --- | --- | --- | --- | --- | --- | --- | --- | --- | --- | --- | --- | --- | --- | --- | --- | --- | --- | --- | --- | --- | --- | --- |
| **0.00** | **1.00** | Gender | | | | | | | | | | | | | | | | | | | | | | | | | | | |
| **0.00** | **0.00** | **1.00** | PTA | | | | | | | | | | | | | | | | | | | | | | | | | | |
| **0.00** | **0.00** | **0.00** | **1.00** | BDI | | | | | | | | | | | | | | | | | | | | | | | | | |
| **0.00** | **0.00** | **0.00** | **0.00** | **1.00** | GÜF | | | | | | | | | | | | | | | | | | | | | | | | |
| **0.00** | **0.00** | **0.00** | **0.00** | **0.00** | **1.00** | PTAnoise | | | | | | | | | | | | | | | | | | | | | | | |
| **0.00** | **0.00** | **0.00** | **0.77** | **0.00** | **0.00** | **1.00** | GDT | | | | | | | | | | | | | | | | | | | | | | |
| **0.00** | **0.00** | **0.00** | **0.00** | **0.00** | **0.00** | **0.00** | **1.00** | AM | | | | | | | | | | | | | | | | | | | | | |
| **0.00** | **0.00** | **0.00** | **0.00** | **0.00** | **0.00** | **0.00** | **0.00** | **1.00** | FM | | | | | | | | | | | | | | | | | | | | |
| **0.00** | **0.00** | **0.00** | **0.00** | **0.00** | **0.00** | **0.00** | **0.00** | **0.51** | **1.00** | DLI | | | | | | | | | | | | | | | | | | | |
| **0.00** | **0.00** | **0.00** | **0.00** | **0.00** | **0.00** | **0.00** | **0.00** | **0.00** | **0.00** | **1.00** | DPT | | | | | | | | | | | | | | | | | | |
| **0.00** | **0.00** | **0.00** | **0.00** | **0.00** | **0.00** | **0.00** | **0.00** | **0.00** | **0.00** | **0.00** | **1.00** | FPT | | | | | | | | | | | | | | | | | |
| **0.00** | **0.00** | **0.00** | **0.00** | **0.00** | **0.00** | **0.00** | **0.00** | **0.00** | **0.00** | **0.00** | **0.55** | **1.00** | SRT | | | | | | | | | | | | | | | | |
| **0.00** | **0.00** | **0.00** | **0.75** | **0.00** | **0.00** | **0.00** | **0.00** | **0.00** | **0.00** | **0.00** | **0.00** | **0.00** | **1.00** | SiN SNR | | | | | | | | | | | | | | | |
| **0.00** | **0.00** | **0.00** | **0.54** | **0.00** | **0.00** | **0.59** | **0.00** | **0.00** | **0.00** | **0.00** | **0.00** | **0.00** | **0.00** | **1.00** | GS | | | | | | | | | | | | | | |
| **0.00** | **0.00** | **0.00** | **0.59** | **0.00** | **0.50** | **0.57** | **0.00** | **0.00** | **0.00** | **0.00** | **0.00** | **0.00** | **0.54** | **0.83** | **1.00** | Stroop A  Stroop index | | | | | | | | | | | | | |
| **0.00** | **0.00** | **0.00** | **0.00** | **0.00** | **0.00** | **0.00** | **0.00** | **0.00** | **0.00** | **0.00** | **0.00** | **0.00** | **0.00** | **0.00** | **0.00** | **1.00** | Emotional Stroop | | | | | | | | | | | | |
| **0.00** | **0.00** | **0.00** | **0.00** | **0.00** | **0.00** | **0.00** | **0.00** | **0.00** | **0.00** | **0.00** | **0.00** | **0.00** | **0.00** | **0.00** | **0.00** | **0.00** | **1.00** |  | | | | | | | | | | | |
| **0.00** | **0.00** | **0.00** | **0.00** | **0.00** | **0.00** | **0.00** | **0.00** | **0.00** | **0.00** | **0.00** | **0.00** | **0.00** | **0.00** | **0.00** | **0.00** | **0.00** | **0.55** | **1.00** | TMT A | | | | | | | | | | |
| **0.00** | **0.00** | **0.00** | **0.00** | **0.00** | **0.00** | **0.00** | **0.00** | **0.00** | **0.00** | **0.00** | **0.00** | **0.00** | **0.00** | **0.00** | **0.00** | **0.52** | **0.00** | **0.00** | **1.00** | TMT index  STM verbal | | | | | | | | | |
| **0.00** | **0.00** | **0.00** | **0.00** | **0.00** | **0.00** | **0.00** | **0.00** | **0.49** | **0.00** | **0.00** | **0.00** | **0.00** | **0.00** | **0.00** | **0.00** | **0.00** | **0.53** | **0.00** | **0.00** | **1.00** |  | | | | | | | | |
| **0.00** | **0.00** | **0.00** | **0.00** | **0.00** | **0.00** | **0.00** | **0.00** | **0.00** | **0.00** | **0.00** | **0.00** | **0.00** | **0.00** | **0.00** | **0.00** | **0.00** | **0.00** | **0.00** | **0.00** | **0.00** | **1.00** | WM verbal  STM visual | | | | | | | |
| **0.00** | **0.00** | **0.00** | **0.00** | **0.00** | **0.00** | **0.00** | **0.00** | **0.00** | **0.00** | **0.00** | **0.00** | **0.00** | **0.00** | **0.00** | **0.00** | **0.00** | **0.00** | **0.00** | **0.00** | **0.00** | **0.00** | **1.00** |  | | | | | | |
| **0.00** | **0.00** | **0.00** | **0.00** | **0.00** | **0.00** | **0.00** | **0.00** | **0.00** | **0.00** | **0.00** | **0.00** | **0.00** | **0.00** | **0.00** | **0.00** | **0.00** | **0.00** | **0.00** | **0.00** | **0.00** | **0.00** | **0.00** | **1.00** | WM visual | | | | | |
| **0.00** | **0.00** | **0.00** | **0.00** | **0.00** | **0.00** | **0.00** | **0.00** | **0.00** | **0.00** | **0.00** | **0.00** | **0.00** | **0.00** | **0.00** | **0.00** | **0.00** | **0.00** | **0.00** | **0.00** | **0.00** | **0.00** | **0.00** | **0.00** | **1.00** | d2 | | | | |
| **0.00** | **0.00** | **0.00** | **0.00** | **0.00** | **0.00** | **0.00** | **0.00** | **0.00** | **0.00** | **0.00** | **0.00** | **0.00** | **0.00** | **0.00** | **0.00** | **0.00** | **0.00** | **0.00** | **0.00** | **0.00** | **0.00** | **0.00** | **0.00** | **0.00** | **1.00** | PF | | | |
| **0.00** | **0.00** | **0.00** | **0.00** | **0.00** | **0.00** | **0.00** | **0.00** | **0.00** | **0.00** | **0.00** | **0.00** | **0.00** | **0.00** | **0.00** | **0.00** | **0.00** | **0.00** | **0.00** | **0.00** | **0.00** | **0.00** | **0.00** | **0.00** | **0.00** | **0.00** | **1.00** | DF  DA auditory | | |
| **0.00** | **0.00** | **0.00** | **0.00** | **0.00** | **0.00** | **0.00** | **0.00** | **0.00** | **0.00** | **0.00** | **0.00** | **0.00** | **0.00** | **0.00** | **0.00** | **0.00** | **0.00** | **0.00** | **−0.56** | **0.00** | **0.00** | **0.00** | **0.00** | **0.00** | **0.00** | **0.00** | **1.00** |  | |
| **0.00** | **0.00** | **0.00** | **0.00** | **0.00** | **0.00** | **0.00** | **0.00** | **0.00** | **0.00** | **0.00** | **0.00** | **0.00** | **0.00** | **0.00** | **0.00** | **0.00** | **0.00** | **0.00** | **0.00** | **0.00** | **0.00** | **0.00** | **0.00** | **0.00** | **0.00** | **0.00** | **0.00** | **1.00** | DA visual |
| **0.00** | **0.00** | **0.00** | **0.00** | **0.00** | **0.00** | **0.00** | **0.00** | **0.00** | **0.00** | **0.00** | **0.00** | **0.00** | **0.00** | **0.00** | **0.00** | **0.00** | **0.00** | **0.00** | **0.00** | **0.00** | **0.00** | **0.00** | **0.00** | **0.00** | **0.00** | **0.00** | **0.00** | **0.00** | **1.00** |

Age

Age

Education

Education

Gender

PTA

BDI

GÜF

PTAnoise

GDT

AM

FM

DLI

DPT

FPT

SRT

SiN SNR

GS

Stroop A

Stroop index

Emotional Stroop

TMT A

TMT index

STM verbal

WM verbal

STM visual

WM visual d2 PF

DF

DA auditory

DA visual

−1 −0.8 −0.6 −0.4 −0.2 0 0.2 0.4 0.6 0.8 1

Supplementary Figure 3. **Spearman bivariate correlation matrix of variables for individuals with tinnitus.** Only correlations with *p-*values smaller than 0.05 are displayed (not corrected for multiple comparisons). Expected positive correlations between SiN and GS, as well as between BDI and THI. Further, there is a positive correlation between THI and the TMT index. *PTA = puretone average, BDI = Beck's Depression Inventory, GÜF = GeräuscheüberempfindlichkeitsFragebogen, SiN SNR = speech-in-noise signal-to-noise ratio, GS = gated speech, STM = shortterm memory, WM = working memory, THI = Tinnitus Handicap Inventory, VAS = visual analog scale.*


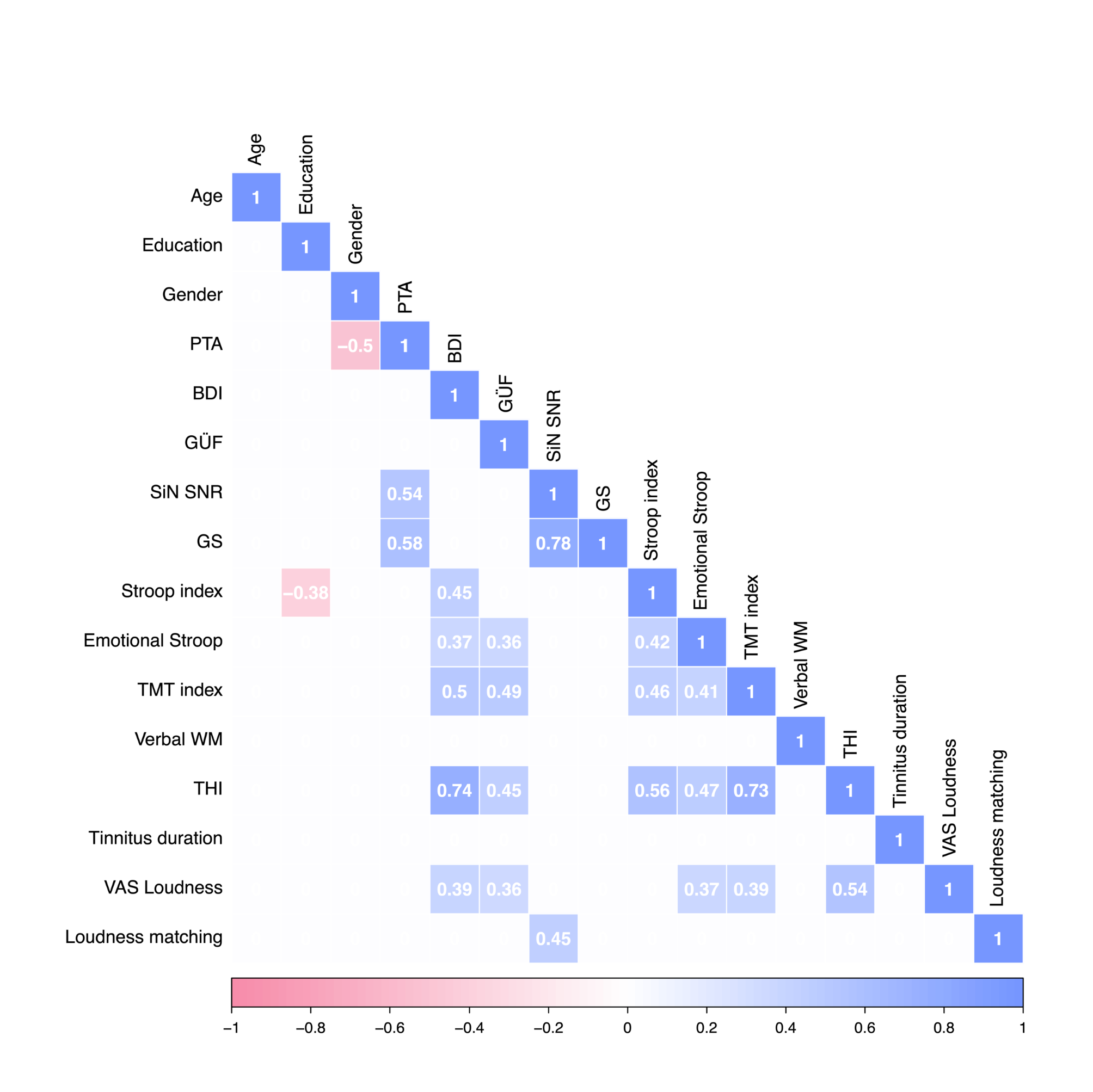


Supplementary Table 1. **Assumptions of the ANCOVA models.** For each individual model, it is listed whether the assumptions of linearity, homoscedasticity, autocorrelation, normality of residuals, and independence are met. Additionally, the table specifies whether any outliers were present and, if so, whether their exclusion altered the results. *PTA = pure-tone average, GDT = gap detection task, AM = amplitude modulation, FM = frequency modulation, DLI = different limen for intensity, DPT = duration pattern test, FPT = frequency pattern test, SRT = speech recognition threshold, SiN SNR = speech-in-noise signal-to-noise ratio, GS = gated speech, TMT = trail making test, STM = short-term memory, WM = working memory, fluency, DA = divided attention, + = fulfilled, - = not fulfilled.*

|  | **Linearity** | **Homoscedas-**  **ticity** | **Autocorrelation** | **Normality of residuals** | **Independence** | **Model** | **Outliers** | | **Change when outliers excluded** |
| --- | --- | --- | --- | --- | --- | --- | --- | --- | --- |
|  | **Residuals vs. Fitted** | **BreuschPagan** | **Durbin-**  **Watson** | **Shapiro-Wilk** |  |  | **Tinnitus** | **Control** |  |
| **PTAwhite noise** | + | + | + | + | + | ANC | 0 | 0 |  |
| **GDT** | + | + | + | + | + | ANC | 0 | 0 |  |
| **AM Average** | + | + | + | - | + | Rank-ANC | 0 | 1 | no |
| **AM 2Hz** | + | + | + | - | + | Rank-ANC | 0 | 1 | no |
| **AM 4Hz** | - | + | + | - | + | Rank-ANC | 1 | 0 | no |
| **AM 8Hz** | - | - | + | - | + | Rank-ANC | 1 | 0 | no |
| **FM Average** | + | + | + | - | + | Rank-ANC | 0 | 2 | no |
| **FM 2Hz** | + | + | + | - | + | Rank-ANC | 0 | 1 | no |
| **FM 4Hz** | + | + | + | - | + | Rank-ANC | 1 | 2 | no |
| **FM 8Hz** | + | + | + | - | + | Rank-ANC | 0 | 2 | no |
| **DLI** | - | + | + | + | + | ANC | 0 | 1 | no |
| **DPT** | + | + | + | - | + | Rank-ANC | 1 | 1 | no |
| **FPT** | + | + | + | - | + | Rank-ANC | 0 | 0 |  |
| **SRT** | + | + | + | + | + | ANC | 0 | 0 |  |
| **SiN SNR** | + | + | - | + | + | Rank-ANC | 1 | 0 | no |
| **GS** | + | + | + | - | + | Rank-ANC | 0 | 0 |  |
| **Stroop A** | + | + | + | - | + | Rank-ANC | 0 | 0 |  |
| **Stroop Index** | + | + | + | - | + | Rank-ANC | 1 | 0 | no |
| **Emotional Stroop** | + | + | + | + | + | Rank-ANC | 2 | 0 | no |
| **TMT A** | + | + | + | - | + | Rank-ANC | 0 | 0 |  |
| **TMT Index** | + | + | + | + | + | Rank-ANC | 0 | 0 |  |
| **Verbal STM** | + | + | + | - | + | Rank-ANC | 0 | 0 |  |
| **Verbal WM** | + | + | + | + | + | ANC | 0 | 0 |  |
| **Visuell STM** | + | + | + | - | + | Rank-ANC | 0 | 0 |  |
| **Visuell WM** | + | + | + | + | + | ANC | 0 | 0 |  |
| **Phonemic fluency** | + | + | + | + | + | ANC | 0 | 0 |  |
| **Design fluency** | + | + | + | - | + | Rank-ANC | 0 | 0 |  |
| **d2** | + | + | + | + | + | ANC | 0 | 0 |  |
| **DA auditiv** | + | - | + | + | + | Rank-ANC | 0 | 0 |  |
| **DA visuell** | + | + | + | - | + | Rank-ANC | 1 | 1 | no |

Supplementary Table 2. **Audiogram comparison between the tinnitus group and the control group for each frequency.** Differences were assessed with a Wilcoxon test or a Student’s t-test, depending on whether or not the assumptions for a Student’s t-test were met. *TI = tinnitus group, CG = control group.*

|  |  |  |  |  | **Frequency (in kHz)** | |  |  |  |
| --- | --- | --- | --- | --- | --- | --- | --- | --- | --- |
|  |  | **0.125** | **0.25** | **0.5** | **1** | **2** | **4** | **6** | **8** |
| **Left** | **TI mean**  **CG mean** | 21.18  19.44 | 12.04  13.03 | 17.72  17.67 | 16.19  12.62 | 22.17  18.93 | 42.23  38.33 | 46.68  37.57 | 57.696  47.417 |
|  | ***p*-Value** | 0.495 | 0.408 | 0.868 | 0.095 | 0.270 | 0.362 | **0.048** | 0.072 |
|  | ***p_bonf_*-Value** | 0.999 | 0.999 | 0.999 | 0.999 | 0.999 | 0.999 | 0.768 | 0.999 |
| **Right** | **TI mean**  **CG mean** | 17.09  18.9 | 9.14  10.5 | 18.46  16.95 | 16.08  16.13 | 25.06  20.74 | 40.49  35.51 | 44.61  38.83 | 58.33  47.37 |
|  | ***p*-Value** | 0.449 | 0.771 | 0.435 | 0.735 | 0.135 | 0.104 | 0.278 | **0.040** |
|  | ***p_bonf_*-Value** | 0.999 | 0.999 | 0.999 | 0.999 | 0.999 | 0.999 | 0.999 | 0.640 |

# Mixed ANOVA

A mixed-design ANOVA was conducted with group (tinnitus vs. control) as a between-subjects factor, and frequency (multiple audiometric frequencies) and ear (left, right) as within-subjects factors, using hearing threshold as the dependent variable. There was no significant main effect of group (*F*(1, 61) = 2.6, *p* = 0.112), indicating that overall hearing thresholds did not differ significantly between the tinnitus and control group. A significant main effect of frequency was found, (*F*(7, 427) = 112.3, *p* < 0.001, Greenhouse-Geisser corrected 𝜀 = 0.3, *p* < 0.001), indicating that hearing thresholds varied significantly across frequencies. There was no significant main effect of ear (*F*(1, 61) = 0.3, *p* = 0.608), and no significant interaction between group and ear (*F*(1, 61) = 0.3, *p* = 0.616). The group × frequency interaction showed a trend (*F*(7, 427) = 2.0, *p* = 0.057, Greenhouse-Geisser corrected 𝜀 = 0.3, *p* = 0.138), but suggesting that the groups did not differ meaningfully. Post-hoc comparisons revealed only one marginal difference between the tinnitus and the control group marginal at 8 kHz (*p* = 0.042, *p_bonf_* = 0.337). A significant frequency × ear interaction was observed, (*F*(7, 427) = 2.3, *p* = 0.029, Greenhouse-Geisser corrected 𝜀 = 0.5, *p* = 0.074). The three-way interaction between group, frequency, and ear was not significant, (F(7, 427) = 0.8, *p* = 0.573).
